# Immune Tumor Microenvironment in Breast Cancer and the Participation of Estrogen and Its Receptors in Cancer Physiopathology

**DOI:** 10.3389/fimmu.2019.00348

**Published:** 2019-03-01

**Authors:** Mariana Segovia-Mendoza, Jorge Morales-Montor

**Affiliations:** Departamento de Inmunología, Instituto de Investigaciones Biomédicas, Universidad Nacional Autónoma de México, Mexico City, Mexico

**Keywords:** immune infiltration, breast cancer, estrogen receptor, estrogen receptor inhibitors, tumor microenvironment

## Abstract

Breast cancer is characterized by cellular and molecular heterogeneity. Several molecular events are involved in controlling malignant cell processes. In this sense, there is an overriding importance to study the multiple cell alterations within this pathology. That the immune response can vary depending on sex is a widely identified fact. Steroid hormones and their receptors may regulate different functions and the responses of several subpopulations of the immune system. Few reports are focused on the function of estrogen receptors (ERs) on immune cells and their roles in different breast cancer subtypes. Thus, the aim of this review is to investigate the immune infiltrating tumor microenvironment and the prognosis conferred by it in different breast cancer subtypes, to discuss the current knowledge and to point out the roles of estrogen and its receptors on the infiltrating immune cells, as well as to identify how different immune subsets are modulated after anti-hormonal treatments in breast cancer patients.

## Introduction

### Breast Cancer and the Microenvironment of Infiltrated Immune Cells

Breast cancer is the most frequently diagnosed malignancy in women worldwide, and it represents the second most common cause of cancer deaths (1). Epidemiological studies have indicated that steroid sexual hormones play important roles in the initiation and progression of breast cancer. Other risk factors are also associated with this disease such as diet, ethnic differences, age, early menarche, not bearing children, having a first pregnancy at over 30 years of age, obesity, genetic mutations, exposure to oral contraceptives, consumption of alcohol or cigarettes, and environmental contaminants, among others. It is estimated that more than 1,000,000 women are diagnosed with breast cancer every year, and more than 410,000 will die from the disease (2, 3). The above indicates that breast cancer represents an important worldwide health problem.

On the other hand, breast cancer is a heterogeneous disease, which is traditionally classified into three phenotypes: luminal [estrogen receptor (ER) positive], human epidermal growth factor receptor type 2 (HER2)-positive, and triple negative (ER-negative/HER2-negative) (4). Moreover, breast cancer is characterized by a highly inflammatory microenvironment, which is supported by the infiltrating immune cells, cytokines, and growth factors (5, 6). In addition, immune infiltration of breast tumors has been shown to be related to clinical outcome through the modulation of treatment response. Breast tumors with immune infiltration are associated with different patterns based on ER presence; however, a common negative immune feature is that regulatory T cells (T regs) are associated with poor prognosis in both ER-positive and ER-negative breast tumors, conferring an immunosuppresive environment (7, 8). Such a feature is a characteristic that highlights the importance of the immune tumor microenvironment in breast cancer.

**Figure 1:**
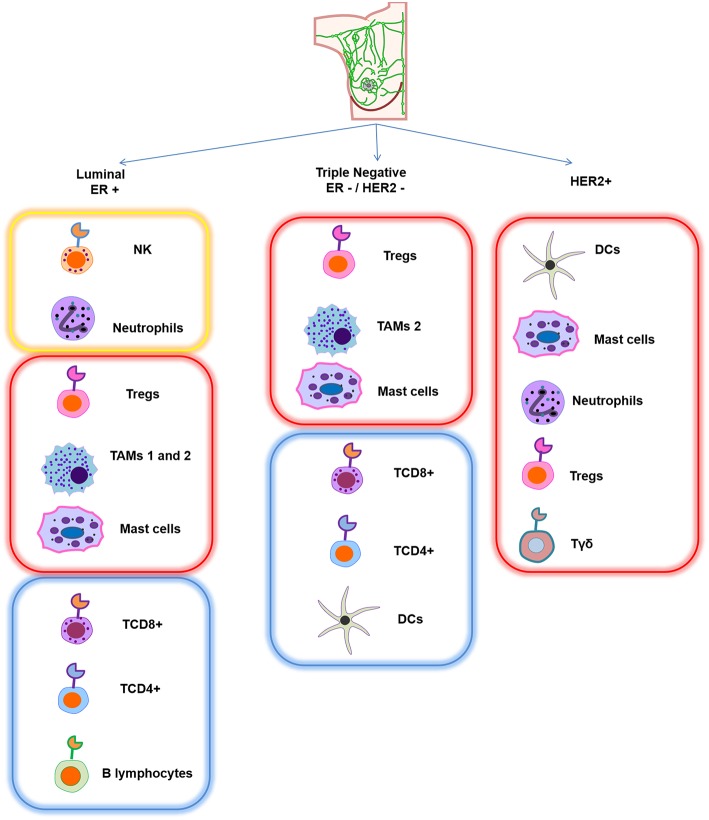
Schematic representation of the main infiltrating immune cell pattern in different breast cancer subtypes. Each subtype has a different composition of immune cells. Yellow frame represents strong presence of specific immune cells that confer good prognosis, red frame indicates that this infiltrating signature is associated with poor prognosis, and blue frame corresponds to a lower proportion of immune cells, which is also associated with good prognosis.

With respect to other infiltrating immune cells in breast cancer phenotypes, a strong proportion of natural killer cells (NK) and neutrophils have been found in ER-positive breast tumors, while cytotoxic T cells (TCD8^+^) as well as naïve and memory T cells (TCD4^+^) are found in smaller proportions. Interestingly, eosinophils and monocytes are associated with a good response after chemotherapy, and B lymphocytes are also associated with good prognosis in this phenotype. Recently, activated mast cells have additionally been correlated with good prognosis (9). However, the presence of this population is still controversial (10). Moreover, in this phenotype, tumor-associated macrophages (TAMs) 1 and 2 and T reg lymphocytes displayed poor prognosis due to their inflammatory, immunosuppressive, and pro-tumorigenic roles (11–14). In ER-negative breast tumors, the major component of immune infiltration cells are T regs, TAM2, and activated mast cells, which are also associated with negative prognosis. In contrast, TCD4^+^, TCD8^+^, B lymphocytes, and dendritic cells (DCs) are related to better prognosis, but they are found in lower numbers and can be associated with a favorable response to neoadjuvant chemotherapy (7, 14–21). With respect to the HER2-positive breast cancer type, there are not many reports about the infiltrating immune mass. However, it is mainly represented by DCs, mast cells, γδ T lymphocytes, T regs and neutrophils—interestingly, all of them confer poor prognosis, disease relapse, and metastasis in this phenotype (see Figure 1) (14, 22, 23).

This intra-tumoral immune pattern establishes a complex relationship between the heterogeneity of immune infiltrating cells, the tumor phenotype, and the treatment response in breast cancer.

## Estrogen Signaling and Estrogen Effects in Breast Cancer Cells

**Figure 2:**
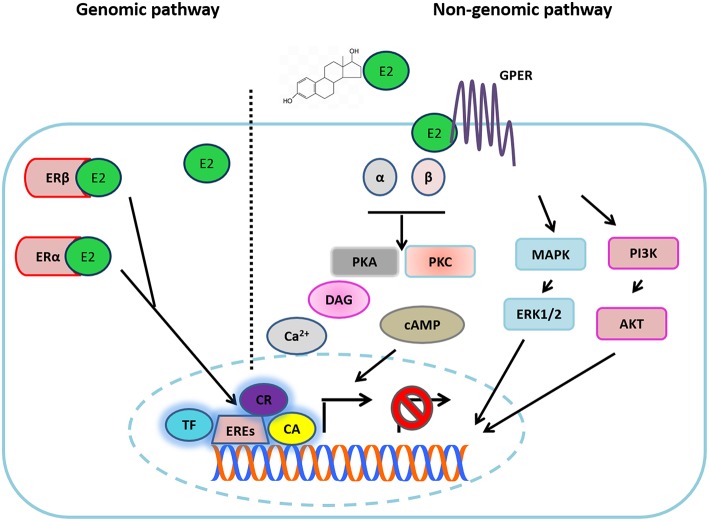
Estradiol signaling. Estradiol (E2) can bind to its different receptors to activate the genomic pathway or the non-genomic pathways. In the first one, E2 binding to ERα and ERβ, each complex is directed to the nucleus where it joins with EREs in the DNA, recruiting different transcription factors (TF), co-activators (CA), or co-repressors (CR) in order to activate or suppress the transcription of target genes. In the non-genomic pathway, E2 binds to GPR30, triggering the activation of G proteins. The above turns out in the increase of different second messengers (cAMP, Ca^2+^, DAG). Additionally, E2 can activate different growth factor receptor (GFR) activity through the non-genomic pathway, which results in the activation of different downstream signaling pathways (MAPK and PI3K) and in the release of different ligands of GFRs.

Estradiol (17β-estra-1,3,5 (10)-triene-3,17-diol) E2 is a steroid hormone produced by theca and a granulosa cell in the ovaries. E2 regulates several physiological and pathological processes, including cancer. Classical or genomic E2 signaling is mainly mediated by two isotypes of the receptor: ERα and ERβ, both of which are nuclear transcription factors that bind to their specific ligand or several estrogens in general; and, subsequently, they form homo- or heterodimers that bind to estrogen response elements (EREs) contained in the promoter region of specific genes in order to activate or suppress their expression. These actions are mediated by the recruitment of distinct co-activators or co-repressors or through the interaction with other transcription factors (Figure 2) (24). E2 actions are also mediated by other non-classical pathways, known as ligand-independent ERα signaling, by a membrane-anchored receptor called G protein-coupled estrogen receptor 1 (GPER1), in which target gene transcription occurs through second messengers and several transcription factors. Thus, GPER1 mediates the increase of different second messengers such as cyclic adenosine monophosphate (cAMP) and diacilglycerol (DAG) levels, mobilization of intracellular calcium (Ca^2+^), and the activation of extracellular signal-regulated kinase (ERK)1/2 and the phosphoinositide 3-kinase (PI3K/AKT) pathways by the trans-activation of the different growth factor receptors (GFRs). Moreover, activation of GPER1 can induce the release of several growth factor ligands such as heregulin, which results in a direct activation of GFRs, depicted in Figure 2 (25–28). It is important to mention that different antagonists or ER inhibitors, such as ICI182,780 and tamoxifen, can mimic the effects of estradiol and induce GPER1 activation.

In breast cancer, E2 can act in different ways. For instance, in immortal cell lines of breast cancer, E2 via ERα signaling is seen to stimulate proliferation, while ERβ activation inhibited cell proliferation and promoted apoptosis (29, 30). Interestingly, estrogen can also undergo several metabolic processes, and its metabolites exert genotoxic effects that contribute to the development of breast cancer through adduct DNA formation (31–33). Many reports on the effects of E2 in breast cancer cells have reported the transcriptional modulation of different genes that are affected; among which are proliferation regulators, growth factors, cell cycle, and apoptotic modulators (29, 34, 35).

Importantly, both classic and membrane ERs have been implicated in several effects of immunity and autoimmunity (36, 37). It is known that the immune system shows remarkable sex-differential responses; thus, this fact potentially suggests that sex hormones such as estrogens address these events. Following this, many reports mention that women respond more aggressively to self-antigens, being more susceptible to autoimmune diseases through of the activation of ER signaling (38). In general, ERs participate in many immune system functions—ERα has been related to spleen and thymus function while ERβ is important for bone marrow functions (24). Both types of ERs are expressed on innate and adaptive immune system cells, indicating an important role for this hormone and its receptor signaling regarding correct immune performance (39).

We describe below the modulation of the most common tumor-infiltrating immune cells by estradiol action upon binding to its receptors in these immune cells of the tumor microenvironment.

## Estrogen Effects on Immune System Cells

### ER in Dendritic Cells (DCs)

DCs are involved in several processes such as immune tolerance, autoimmunity, stimulation, and differentiation of naïve T cells. They are considered as potent antigen presenting cells (APCs) and are mainly activated by stress or damage signs from pathogens that are recognized mainly by Toll-like receptors (TLRs). Following their stimulation via TLRs, DCs secrete pro-inflammatory cytokines to stimulate T lymphocytes and initiate innate immune response. In this sense, ER participates in the favoring of DC function. These cells contain the presence of ERs; when its ligand binds to ERs in these cells it can trigger migration and activation processes. In addition, in mouse *in vitro* models of DCs, estrogen can induce differentiation, survival, and increase the expression of co-stimulatory molecules (39). It has been reported that pre-treatment of E2 in co-cultures of mature DCs with T cells resulted in the stimulation of T cell proliferation (40). Besides, E2 up-regulates the expression and secretion of different pro-inflammatory cytokines and chemokines such as tumor necrosis factor alpha (TNFα), interleukin (IL)-6, CXCL-8 (IL-8), and monocyte chemo-attractant protein 1 (MCP-1) (40). This concept can be directly related to the improvement of DCs' capability to mediate the presentation of self and foreign antigens, and, potentially because of this, the immune system response against tumors is better in early stages of the disease. Nevertheless, the presentation process is disrupted by E2, since after hormone exposure, production of INF-γ and IL-2 is decremented in mature DCs (41). This suggests that the effects of E2 in DCs depend on their maturation stage. Thus, it would be interesting to determine the degree and phenotype of DC maturation in tumors. In addition, differentiation of functional DCs from bone marrow can also be modulated by this hormone since it favors their migration to lymph nodes, an effect that was reverted with the use of specific ERα antagonist (ICI 182,780) (42–44). Supporting this notion, E2 induces myeloid DC differentiation through the activation of two inflammatory-related proteins, the interferon regulatory transcription factor 4 (IRF4) and the participation of granulocyte macrophage colony stimulating factor (GM-CSF). Interestingly, it was reported that the exacerbated activation of these two factors by E2 at some point can lead to a tolerogenic phenotype for DCs (45). The association of ERα with other proteins such as thiolase and glutathione S-transferase P (GSTP) is also linked with DC differentiation. In addition to this, metabolic function, several growth factors, and accessory proteins in bone marrow derived from mice DCs are also affected. On the contrary, the absence of GSTP enhanced DCs' metabolism, their proliferative and differentiation rates, and their effector functions (46). It is important to note that not only does E2 have effects in DCs, an estradiol metabolite, estriol also generated tolerogenic DCs in an *in vivo* model that protects against autoimmunity (47). The above highlights the need to monitor the effects of ER inhibitors on different immune cell functions, favoring not only the inhibition of cancer cells but also the migration of the immune cells to lymph organs or avoiding their anergic phenotype.

### ER in Macrophages (Mø)

Macrophages are a fundamental part of the innate defense mechanisms against foreign pathogens, and they can promote specific immunity by inducing T cell recruitment and activation. Their role is essential for triggering adaptive immune response. Macrophages collaborate with T and B cells based on the release of cytokines, chemokines, and reactive radicals, among other proteins. Despite this fact, their presence within the tumor microenvironment has been associated with enhanced tumor progression and promotion of cancer cell growth, angiogenesis, and immunosuppression (11, 48).

Several articles have reported the presence of ER in monocytes and macrophage precursor cells (49, 50), that the expression of this hormone receptor varies between stages of differentiation, and that monocyte expresses ERβ while macrophages express ERα (51). Recently, however, both receptors have been found in macrophages (52). E2 treatment has been shown to modulate different macrophage actions and their metabolism; for example, it is well-known that production of nitric oxide (NO) into the macrophages allows them to exert antimicrobial and antitumor actions (53). Related to this concept, hormone treatment stimulated NO release in human peripheral monocytes and in a murine macrophage cell line via GPER activation coupled with intracellular calcium influx (54, 55). In line with this, stimulation with LPS in isolated peritoneal macrophages from young female rats resulted in elevated NO release; this effect was not observed in macrophages derived from the middle-aged animals, where circulating E2 levels were diminished (56). Moreover, macrophages produce and use arachidonic acid and its different metabolites for the recognition of pathogens and to enhance or suppress inflammatory response (57). E2 has been shown to modulate the lipid metabolism of macrophages since it elicits an increase of arachidonic acid release and prostaglandin E2 production (a derivative of arachidonic acid) in human monocytic cell lines (58). In addition, the phagocytic activity of macrophages is performed in part by reactive oxygen species (ROS)—which cause DNA or cell membrane damage—and the interplay between intracellular ROS and antioxidant enzymes such as superoxide dismutase (SOD), catalase (CAT), and glutathione peroxidase (GSH-Px) is important in the macrophage phagocytic function, activation, differentiation, and recruitment process (59). In this context, it has been reported that E2 administration in rats modulated CAT activity in *ex vivo* macrophages (60). Part of the bacterial killing mechanism of macrophages induced by LPS is the activation of metalloproteinases (MMPs); gene expression of MMP-9 especially was dramatically reduced after E2 treatment in rat cell lines of macrophagic origin, and this effect was blocked with ICI 182,720 treatment (61). This hormone also modulates macrophage survival, and this effect was reported in an *in vitro* culture of human macrophages where E2 treatment induced the anti-apoptotic protein Bcl-2, action mediated by the modulation of the intracellular Ca^+2^ concentration, the activation of protein kinase C, and ERK phosphorylation (62, 63). Furthermore, macrophages can recognize distinct pathogen-associated molecule patterns (PAMPs) which contributes to activating several signaling cascades and diverse cytokines and chemokines (64). E2 via ERα reduced gene and protein expression of the pro-inflammatory IL-8 in monocytes previously challenged by LPS (65). The modulation of this chemokine impacts not only the macrophage's function but also the neutrophil's recruitment to inflammation sites, mediating pathogen clearance (66). E2 can also modulate other functional macrophage cytokines; its treatment decreased IL-6, TNF-α, and IL-1β expression in whole blood cultures derived from healthy postmenopausal women, in bone marrow cell cultures, and in *ex vivo* rat macrophages (56, 67–70). The modulation of these cytokines was confirmed to be an E2-dependent effect, according to the opposite event found in these cells when they were treated with ICI 182,780 (69). A similar result from E2 treatment related to the decreased expression of the TNF-α gene was reported in an ER-positive murine monocytic cell line through of the down-regulation of Jun NH (2)-terminal kinase activity, with a consequent decrease of AP-1 transcription factor, affecting TNF-α transcription (71). In addition, E2 modulates the macrophage's activation (72), which is mainly classified into two categories: classical activation (macrophages kill microbes and act as anti-tumor effector cells), which is promoted by IFN-γ, TNF-α, and TH2-related cytokines or alternative activation (macrophages lay down extracellular matrix components to promote wound healing, angiogenesis, and sustain tumor progression). This type of macrophage activation is promoted by TH1 cytokines, being an IL-4/IL-13-dependent mechanism (73). The effect of this hormone in macrophage activation was clearly observed in a murine wound healing model in ovariectomized mice. In this sense, macrophages coming from ovariectomized animals show preferentially a classical activation. In addition, the gene expression of two alternative activation macrophages markers (Fizz1 and Ym1) was reduced, and the ovariectomized mice also presented a reduction in both macrophage numbers in the wound area and the inflammatory environment through the reduction of monocyte-associated TNF-α secretion as compared with the intact group. In contrast, E2 supplementation in ovariectomized mice restored the expression of both markers, leading to alternative macrophage activation, wound repair, remodeling, and angiogenesis (72). Furthermore, the alternative macrophage activation promoted by E2 has been documented in other assays. With respect to this notion, the gene expression of arginase 1, another established alternative activation macrophage marker, was up-regulated with ERα agonist treatment in an *in vitro* culture of bone marrow-derived macrophages (74). This work also evaluated the role of E2 in wildtype or in mice with ERα and inflammatory gene deletion (LysM-ERα) subjected to incisional wounds with a subsequent exogenous E2 replacement. Of note, in the absence of the hormone, healing was delayed (74) as has been previously reported in an ovariectomized wildtype mouse model (72). However, the hormone treatment revealed increased recovery in healing response, whereas in ERα knockout mice it resulted in a marked healing delay. The above highlights the role of estradiol-ERα action in the induction of alternative macrophage activation (74). Additionally, the role of E2 in favoring alternative macrophage activation was corroborated in an *in vitro* and *ex vivo* study on human blood-derived macrophages. In fact, classical lipopolysaccharide (LPS)/IFN-γ stimulus on un-polarized macrophages induced the down-regulation of two markers of alternative activation (CD163 and CD206); these effects were avoided through treatment via the modulation of NFκB transcription factor (75). Interestingly, much evidence supports the notion that macrophages, especially alternatively activated macrophages, shape immune tumor infiltration and have influence in high vascular grade associated with metastasis (76–79). In this sense, breast cancer phenotype can also regulate the type of infiltrating macrophage phenotype (80). Current evidence suggests that this population of macrophages regulates at the same time ERα expression in an epigenetic manner through the modulation of a DNA hydroxymethylation marker, ten-eleven-translocation 5-methylcytosine dioxygenase (TET1). The above was demonstrated in co-cultures of endometrial cancer cells with alternatively activated macrophages, with the results showing that alternatively activated macrophages enhanced both E2-driven endometrial cancer cell proliferation and up-regulation in ERα expression, a mechanism dependent on IL-17A expression (81). The above highlights the importance of the interplay among sex steroids, the immune system, and tumor progression.

### ER in Mast Cells

Mast cells (MCs) are tissue-resident immune cells that form part of the innate immune system. They are commonly associated with allergic reactions and parasitic infections. These cells are characterized by the presence of granules loaded with different inflammatory mediators that they release depending on the time and the type of stimulus (82). Additionally, secretion of serine proteases such as tryptase or chymase define what phenotype of the mast cell will be activated, which means that mucosal mast cells produce tryptase and the connective tissue mast cells secrete tryptase, chymase, and carboxypeptidases (83). These enzymes—in conjunction with the release of IL-8, tumor growth factor beta (TGF-β), and TNF-α–have been associated with angiogenesis trough vascular endothelial growth factor (VEGF) and MMP modulation in different breast cancer phenotypes (9, 84). Mast cells can be activated by the direct recognition of pathogen-associated molecular patterns (PAMPs) or by immunoglobulins and immunoglobulin E receptor (Fcϵ RI) interaction; both cases result in the release of different molecules from their granules, recruiting different immune cells.

On the other hand, several studies have reported the presence of ERα but not ERβ in mast cells; however, it was recently described that these cells have the presence of both nuclear receptors (85–88). In this sense, treatment of E2 or an endocrine disrupting compound such as bisphenol A has been demonstrated to induce the release of histamine (an important biomolecule involved in allergic reactions) from rat mast cells in a concentration-dependent manner (89). Of note, the histamine release is also important in breast cancer promotion since this protein or its receptors (H3R and H4R) have been associated with the induction of breast cancer cell proliferation and migration. Importantly, these molecules have been identified to a greater extent in breast tumor samples as compared with non-tumor samples (90). The above suggests that the inhibition of this molecule could result in an interesting target in this disease. E2 has an important role in inducing the release of asthma mediators such as leukotriene and β-hexosaminidase in a rat mast cell line. The release of β-hexosaminidase has also been described in both the human mast cell line and in a primary culture (non-transformed) of mast cells. This action was blocked with the addition of tamoxifen or ICI 182,780, demonstrating that ERα is responsible for these actions (89, 91, 92). In relation to breast cancer progression, tryptase release from mast cells has been closely associated with an increased number of carcinoma-associated fibroblasts in breast tumor samples, favoring the tissue remodeling and angiogenesis (93). Related to this, E2 up-regulates tryptase secretion in the human mast cell line HMC-1 (88), assuming that it induces the degranulation of these cells. In addition, E2 in an *ex vivo* model induces the expression of two chemokine receptors (CCR4 and CCR5), which are implicated in the migration of periphery mast cells to the uterus (88, 94). The above highlights the effects of E2 in mast cell function with the purpose of favoring breast cancer progression. On the other hand, there are few reports with respect to E2 function by the non-genomic pathway in mast cells. In this regard, it has been shown that estradiol induces the release of intracellular calcium, which is important for degranulation and leukotriene synthesis in mast cells (95). Recently, the role of mast cells in breast cancer has been largely studied (10); however, many of their functions and components in their granules in relation with breast cancer progression are still little addressed, and this makes them an important population for study in the cancer microenvironment.

### ER in Neutrophils

Neutrophils, which are other fundamental pathogen-fighting immune cells, constitute the first line of host defense. They can be recruited to infection sites and eliminate microbes by classical phagocytosis or degranulation, and they also produce ROS, release antimicrobial peptides, or expel their nuclear content in order to form neutrophil extracellular traps (NETs) (96, 97). Neutrophils collaborate with other immune cells such as macrophages or DCs and secrete many chemokines and cytokines that regulate the immune response (98). It has been described that neutrophils as well as other immune cells present both nuclear receptors (99). In this regard, E2 through ERα binding has been shown to regulate neutrophil survival, function, and number. E2 exposure delayed apoptosis in human neutrophils, and this effect was correlated with a significant decrease in active caspase 3 protein expression and was reverted by ICI 182,780 treatment (100). This represents a possible explanation of sexual dimorphism, being that neutrophil number differs between men and women (101). One effect of E2 on the function of neutrophils is that it enhances NO production and nitric oxide synthase, demonstrated previously in human neutrophils (102, 103). Additionally, neutrophils secrete several serine proteases (NSPs), including neutrophil elastase (NE), proteinase 3 (PR3), and cathepsin G (CG), which are essential for the elimination of infectious agents and the modulation of inflammation (104). Neutrophils derived from splenocytes of mice administered with E2 showed incrementation of NE, PR3, and CG in gene and protein expression as compared with placebo-treated mice. Moreover, E2 administration in these mice increased the number of neutrophils in different lymphoid tissues (splenocytes, peripheral blood, and bone marrow) and the gene and protein expression of myeloperosidase, a major component of neutrophil granules (105). E2 via ERα modulated inflammation, and the actions mentioned above were associated with an autoimmune disease as an increase in neutrophil number and NSPs were found in mice with lupus (105). Moreover, G1-GPER1 activation also participates in neutrophil polarization (analogous concept of macrophage activation) (106), promoting the gene expression of the pro-inflammatory phenotype (N2) and its lifespan, actions mediated by the activation of the cAMP/PKA/CREB, MAPK, and p38 signaling pathways (107). This work also shows that IL-1β, IL-8, the prostaglandin-endoperoxide synthase (PTGS2), the suppressor of cytokine signaling 3 (SOCS3), and granulocyte colony-stimulating factor (G-CSF) gene expression, were enhanced after stimulation of G1-GPER1 in a dose-dependent manner. Additionally, the release of IL-8 was significantly increased as compared with non-treated human neutrophils and with neutrophils stimulated with LPS. Furthermore, this hormone–receptor interaction up-regulated the surface expression of two markers of neutrophil activation (CD11b and CD62L) (107), supporting the notion that G1-GPER1 interaction is responsible for IL-8 neutrophil release. Other work proved that 17β-estradiol-ERα did not induce the release of this chemokine; in fact, the estradiol treatment had the opposite effect in the release of this chemokine in human neutrophils pre-stimulated with LPS (108). In addition, this classical activation may participate in the attenuation of neutrophil activation. E2 reduced the shedding of a surface adhesion neutrophil molecule (CD62L or selectin) (108), which is normally implicated in diapedesis at sites of tissue injury and inflammation (109). Also, E2 treatment blocked the neutrophil chemotaxis promoted by IL-8, and the generation of superoxide anion by neutrophils was diminished with this hormone treatment (108, 110, 111), affecting their host defense function (112).

It is well-known that a certain type of breast cancer is dependent on E2 action; coupled with this notion, this hormone can promote inflammation through the induction of neutrophil infiltration and the expression of pro-tumoral cytokines/chemokines and tissue-remodeling enzymes in mammary neutrophils (113). In a mammary involution mice model, E2 administration induced mammary neutrophil infiltration and neutrophil pro-tumoral activity signature, as at least 10 inflammatory genes were up-regulated in mammary resident cells; interestingly, neutrophil depletion reversed the expression pattern of these inflammatory genes. Moreover, in this mammary involution mice model, the mice were administrated with E2 and injected with a triple negative breast cell line (4T1). Again, the hormone treatment induced mammary neutrophil infiltration—however, neutrophil depletion with a specific antibody resulted in the marked abolition of estrogen-induced mammary tumor growth (113). The mammary neutrophil recruitment induced by this hormone was observed in other *in vitro* and *in vivo* breast cancer research, in which it promoted N2-neutrophil polarization, correlated with the overexpression of integrin LFA-1 and TGF-β, intra- and extravasation and trans-endothelial breast cancer cell migration, and with major breast tumor growth; this last effect was reversed by ICI 182,780 treatment. In fact, E2 treatment transformed a non-metastatic breast cancer cell line into one that was metastatic-associated in the presence of neutrophils (114). The previous observations provide the presence of mammary neutrophils and its activity—which are importantly regulated by E2—with a significance regarding cancer progression.

### ER in NK Cells

NK cells are central components of the innate immunity and they participate in preventing and controlling infections, tumor growth, and metastasis (115). Usually, in tumors there is a downregulation of self-ligands and expression of stress-induced ligands which can be recognized by NK cells (116). Their activation also leads to secretion of stimulatory cytokines and chemokines such as IFN-γ, TNF-α, GM-CSF, MIP1-α, and RANTES, which participate in the stimulation of the adaptive immune system. Moreover, their biological importance lies in their ability to exert a cellular cytolytic effect through the liberation of granzymes and perforin (117).

Since the 1990s, it has been known that E2 causes a reduction in NK cell cytotoxic activity in mice models in a dose-dependent manner (118, 119). This data was confirmed when the hormone was administered in postmenopausal and premenopausal women, resulting in a reduction in NK cell activity (120). In fact, the use of oral contraceptives, which bind to sex steroid receptors, has been associated with changes in NK cytotoxic activity and with an increase in infections (121). Interestingly, the suppressive effect of E2 on the NK cells was attributed to the enhancement of metastasis in a fibrosarcoma and melanoma cell model, where immunosuppressed mice treated with this hormone also exhibited deficient NK cell activity and increased susceptibility to develop metastasis of allogeneic tumor cells (122). Additionally, synthetic non-steroidal estrogens such as diethylstilbestrol showed the same effects regarding inhibitory NK cell activity and the mice's susceptibility to generating tumors derived from this NK cellular inhibition. Of note, NK inhibitory activity was dramatically affected with only neonatal administration of diethylstilbestrol into the mice (123). On the other hand, it has been described that E2 can induce or suppress NK cell activity in mice, with the actions being dependent on time. At short time intervals it acts in a stimulating way, and at long time intervals it suppresses NK cell activity (124). Estrogen can also inhibit NK cell-mediated apoptosis due to the fact that this hormone induced a granzyme inhibitor, named proteinase inhibitor 9 (PI-9) (125). Today, there are few reports that evaluate the effects of E2 in NK cells. However, it is known that the reduction of their activity is related to the promotion of tumor growth (126); therefore, NK cells might be considered as a target for immune therapies in order to avoid the estrogen-mediated increase in breast tumor incidence.

### ER in B Cells

B lymphocytes are part of the adaptive immune system that is specialized in antibody production, which is part of humoral immunity (127). It has been described that B lymphocytes have the expression of both nuclear ERs in all B cell subsets (39, 128). In this sense, E2 has stimulatory effects on B-differentiated lymphocytes derived from human PBMCs. It increased immunoglobulin (Ig)G and IgM production in a dose-dependent manner, and this effect was enhanced by the addition of IL-10, an anti-inflammatory cytokine, to B cells previously treated with E2 (129), and the above becomes relevant in an autoimmune context. The stimulatory effect of E2 on antibody titers has been observed since the 1980s in *in vitro* studies and in the serum of rats administered with this hormone, where an increase in IgM antibodies was reported (130, 131). Of note, it has been reported that IgMs have a direct cytotoxic effect on transformed cells through the activation of the complement pathway (132, 133). This is relevant since the increases on IgMs levels due to E2 exposure are important for breast cancer suppression. Besides, they also might serve as diagnostic indicators of the phenotype or stage of this pathology due to the fact that they are well-correlated with the clinical score and disease spread of breast cancer patients (134); however, more studies are necessary to confirm this fact. Added to that, E2 through the ERα pathway also impacts the activation and survival of B cells through the modulation of several genes. These effects were observed in splenic B cells derived from ovariectomized mice (or not) administered with it. Interestingly, these results were reverted in mice treated with ICI 182,780 (128). Regarding the effects of GPR30 on B lymphocytes, some reports have mentioned that different chemokines can activate it, triggering different roles of B subsets such as migration, chemotaxis, proliferation, and apoptosis, among others. In fact, this receptor has been correlated with different B cell malignancies such as leukemia and lymphomas (135, 136). Nevertheless, more information or mechanisms of action related to this topic would be interesting in relation to the pathogenesis of breast cancer.

### ER in TCD4^+^ and TCD8^+^ Cells

Lymphocytes have important roles in immune protection; traditionally, these cells are divided into two subtypes, TCD4^+^ and TCD8^+^. The first subtype can help B cells to produce antibodies, in order to induce immune response through activation of macrophages and recruitment of different immune cells to specific sites with inflammation. The second type is important for defense against cellular pathogens, among other functions. These immune populations can contribute to attenuate inflammation, production of antibodies, and protection of pathogens (137). Based on the different cytokine secretion profiles, TCD4^+^ is divided into different subsets—for instance, T helper (Th)1 and Th2. Th1 is characterized by secretion of INF-γ, IL-2, IL-12, and TNFα, which are cytokines that stimulate macrophages' functions and cellular response; meanwhile, Th2 cells secrete IL-4, IL-5, IL-6, and IL-10, which are important cytokines for B cell antibody production and humoral response.

It has been described that E2 affects the size, maturation, and development of T cells, a process known as thymic atrophy (37, 138), and this effect is mainly caused by ERα signaling (139). Moreover, it can also influence the expression of the phenotype of CD4^+^/CD8^+^ T cells and their subsets' functions (140), and it also contributes to the development of other T cell subtypes from the lymph nodes, such as Th17 cells (141). Interestingly, the proliferation and generation of active T cells are governed by different metabolic glycolytic demands (142). In this sense, the orphan nuclear hormone receptor, estrogen-related receptor-α (ERRα), is a key regulator that supports T cell functions, since the inhibition of ERRα decreased several glycolytic genes implicated in inflammatory cytokine production and T cell proliferation in an *in vitro* and in an experimental autoimmune encephalomyelitis mouse model, and a similar effect was found in ERRα-deficient T cells (143). Several studies have demonstrated that E2 modulates IFNγ-secretion of Th1 cells in both human and mice cells, which is potentially mediated by direct interaction of ER with its EREs in the promoter region of the IFNγ gene (38, 144, 145). This cytokine has a pivotal role against intracellular infections as well as autoimmune and inflammatory disorders. Furthermore, E2 inhibits the production of Th1 pro-inflammatory cytokines such as IL-2, IL-12, IFN-γ, and TNFα (146). In accordance with this notion, the decline of ovarian function related to menopausal state in women and reduction in the production of this hormone have been associated with an increment in pro-inflammatory cytokine production (147). In line with that, Th1-related cytokine levels such as IL-2 and IFN-γ were augmented in postmenopausal women, and hormone replacement therapy in this population caused a significant decrease of these cytokines (148). On the other hand, the effects of E2 in Th2 cells are related to the increment of anti-inflammatory cytokines such as IL-10, IL-4, and TGF-β (146, 149). In addition, IL-4 incrementation has been correlated with the increase of an essential Th2 transcription factor (GATA-3) (150). Interestingly, E2 administration in a mammary involution mice model diminished CD4^+^ and CD8^+^ T cells in mammary tissue, highlighting the effects of this hormone on the function of these immune cells' type (113).

### ER in Regulatory T Cells (Tregs)

Tregs are involved in self-tolerance, suppression of immune cell functions, down-regulation of self-reactive lymphocyte action, and prevention of transplant rejection through activation of a lineage-specific transcription factor that governs Treg development, differentiation, maintenance, and function—forkhead/winged helix transcription factor (FoxP3) (151). The T regs' immunosuppressive T cell inflammatory activity includes IL-10 secretion and the induction of programmed cell death 1 receptor (PD-1) (137, 152). In breast cancer, these cells are associated with a high rate of relapse and with favoring the tumor microenvironment (7, 16).

E2 *in vitro* and *in vivo* mouse models have been shown to induce the gene expression of FoxP3 and IL-10. These effects were reversed with the treatment ICI 182,780 (153, 154). It also modulates the Tregs' inhibitory capacity, since estradiol treatment increased intracellular PD-1 levels in Tregs coming from splenocytes of wildtype mice, whereas an opposite effect was seen in ER knockout mice (155). E2 treatment has also been shown to promote the proliferation and the number of human Tregs. In addition, it favors the change of CD4^+^, CD25^−^ cells to a CD4^+^, CD25^+^ Treg phenotype (156). Interestingly, a recent work demonstrated that infiltrating Tregs derived from human cervical cancer contain elevated levels of estrogen. Additionally, E2 through ERα signaling binds in the EREs of the Tregs' FoxP3 promoter. In this way, a loop is formed and leads to the activation of FoxP3 activity (157). As in other works, ICI 182,780 treatment reverted effects of E2 in Tregs and resulted in the ablation of FoxP3 protein expression and a decrease in TGF-β secretion (157). Another study supports the notion that in addition to ERα signaling, GPER with the estrogenic small molecule (G-1) is critical for the expansion of Tregs and the induction of the Foxp3 protein in *ex vivo* cultures of purified TCD4^+^ mouse cells. In addition, G-1-GPER activation was able to maintain the Tregs' phenotype and to induce the expression of two proteins implicated in the control of immune homeostasis, PD-1, and cytotoxic T lymphocyte-associated protein 4 (CTLA-4) in the presence of Th17 cytokine inflammatory polarization conditions (158). It has been described that Tregs secrete immunomodulatory cytokines such as TGF-β and IL-10 (159). This cytokine secretion pattern was favored with E2 treatment in Treg cells isolated from peripheral blood mononuclear cells (PBMCs) of healthy women (160). The previous data highlight the fundamental role of the estradiol-ERα / G1-GPER pathway in Treg physiology.

**Figure 3:**
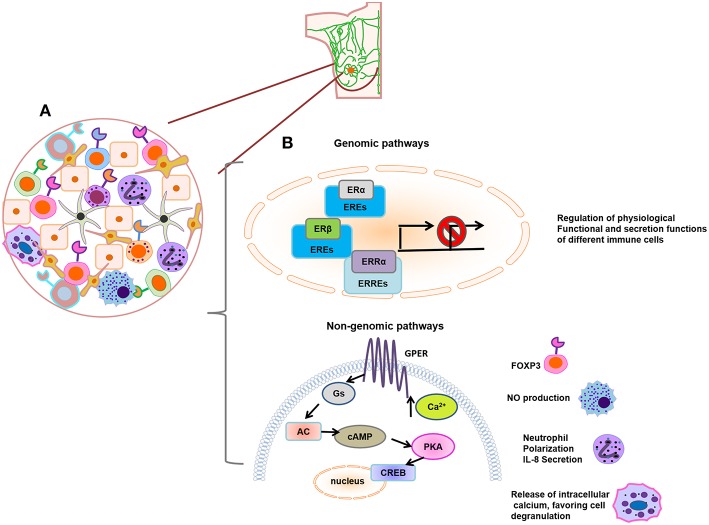
**(A)** Schematic representation of immune infiltrating tumor cells. **(B)** Genomic and non-genomic estrogen pathways on immune cells. Estrogen regulates the physiological, functional, and secretion actions of different immune cells; these effects are mainly studied by the activation of genomic pathways such as ERα, ERβ, or ERRα. In addition, little effects of Tregs (increase of Foxp3 expression), macrophages (NO production), neutrophils (neutrophil polarization and IL-8 secretion), and mast cells (mobilization of intracellular calcium, favoring cell degranulation) have been described by the action of non-genomic pathway.

The estrogen pathways on immune cells studied in the basal condition described above are illustrated in Figure 3, which highlights that few reports have evaluated the effects of Tregs, macrophages, neutrophils, and mast cells mediated by non-genomic pathways. It also aims to represent how immune infiltration is found in breast cancer. In addition, Table 1 summarizes the effects of estrogen on the immune cells that we described in the previous section.

**Table 1:** Estradiol effects of different immune cells.

**Type of immune cells**	**Modulation**	**Reference**
DCs	Increase expression of co-stimulatory molecules such as INF-γ Stimulation of T-cell proliferation and differentiation Induction of pro-inflammatory cytokines and chemokines; TNFα, IL-2, IL-6, IL-10, IL-8, MCP-1 DCs migratory response to lymph nodes after LPS stimulation Induction of DC differentiation via GM-CSF and the IRF4 Generation of tolerogenic DCs affecting their cell antigen presenting function	(39–47)
Macrophages	Stimulate NO release Modulate the lipid metabolism of macrophages through the release of arachidonic acid and prostaglandin E2 production Modulate catalase CAT activity Reduce MMP-9 expression Increase macrophage survival through Bcl-2 activation Reduce IL-8 expression Decrease IL-6, TNF-α, IL-1β expression Reduce TNF-α gene expression Induce alternative macrophage activation through the modulation of activity and expression of several markers such as Fizz1, Ym1 and arginase 1, CD163 and CD206	(49–81)
Mast cells	Induction of histamine, leukotriene, β-hexosaminidase and tryptase release Induction of chemokine receptors (CCR4 and CCR5) Release of intracellular calcium favoring degranulation	(10, 85–94)
Neutrophils	Enhance NO production and the neuronal nitric oxide synthase Promote neutrophil pro-inflammatory phenotype through GPER- cAMP/PKA/CREB, MAPK activation Increase IL-1β, IL-8, PTGS2, SOCS3, and G-CSF gene expression Increase IL-8 release via G1/GPER Up-regulation of two markers of neutrophil activation (CD11b and CD62L) Reduce IL-8 neutrophil release and CD62L expression via ERα Reduce neutrophil chemotaxis and superoxide anion production Increase the number of neutrophils in different lymphoid tissues and the NSPs including NE, PR3, and CG Increase MPO expression	(100–114)
NK cells	Reduction of NK cells' cytotoxic activity over long period of exposure Enhancement of tumor susceptibility and metastasis Stimulation of NK cell activity in short period of exposure Induction of PI-9	(118–126)
B lymphocytes	Enhancement of IgG and IgM production Increase survival, proliferation, migration, and chemotaxis	(39, 128–136)
TCD4^+^ and TCD8^+^Th1Th2	Promotion of CD4^+^ /CD8^+^ T phenotype expression Induction of glycolytic genes implicated in the inflammatory cytokine production and T cell proliferation via ERRα Inhibition of pro-inflammatory cytokines IL-2, IL-12, IFN-γ, and TNF-α Negative regulation of IFNγ promoter Increment of IL-10, IL-4, and TGF-β Induction of Th2 transcription factor GATA-3	(37, 38, 113)
Tregs	Induction of FoxP3 and IL-10 gene expression Maintenance of Tregs phenotype Activation of FoxP3 activity via estradiol-ERα-EREs Induction of FoxP3, PD-1, and CTLA-4 protein expression via GPER Increase of immuno-modulatory cytokines such as TGF-β and IL-10	(153–160)

## Regulation of Immune Cell Functions by ER Inhibitors Treatment in Breast Cancer Patients

It is widely known and accepted that the use of inhibitors of ER in the treatment of patients with estrogen-positive breast cancer has offered high survival rates (161). However, their use in other breast cancer phenotypes and their effects on the immune system cells in clinical stages have not been addressed.

In the previous section, we described *in vitro* and *in vivo* data that clearly show how the tumor infiltrating immune cells could play an important role in the development, progression, and response of breast cancer through ER signaling activation. However, they also encourage focusing on the modulation of their antitumor functions with ER inhibitors. In this regard, few studies have reported the effect of ER inhibitors on immune infiltrating breast tumor cell functions in clinical phases. As we described previously, Tregs have been found to be up-modulated in breast tumors, and a high number of these cells were present in high-grade ER-negative breast cancer patients. Also, they were associated with ER-positive breast tumors identified with high-risk patients (7, 162). It is known that Tregs give valuable information about breast cancer prognosis and progression, since a high number of Tregs can identify patients at risk of relapse after 5 years. Nevertheless, there was no relationship between the number of Tregs and the type of therapy that patients received (7). Interestingly, in 2009, Generali et al. reported that the number of Tregs was significantly decreased in patients who received an aromatase inhibitor treatment alone (letrozole) and in combination with an antineoplastic agent (letrozole + cyclophosphamide) (163). Another *in vivo* model reported that ICI 182,780 could reverse the estradiol actions for inducing Treg phenotype (154). These facts possibly indicate that E2 inhibition is an important antitumor strategy for manipulating the tumor microenvironment through inhibiting the function and number of Tregs; additionally, letrozole might also be useful in combination treatments in patients with ER-negative tumors regardless of ER expression in the tumor cells. Returning to the fact that this hormone can inhibit NK activity, an interesting work reported that post-menopausal stage I breast cancer patients who received tamoxifen for 1 month showed a statistically significant increase in NK activity; however, NK activity could not be related to ER expression in breast tumors due to the limited number of patients included (164). This fact correlates with mice models and estrogen actions in NK cell activation (124). It is also important to mention that some studies have reported a low proportion of NK cells in late stages of breast tumors (165); therefore, the work of Berry et al. suggests that in the early stages of breast cancer, patients treated with tamoxifen could benefit from the activation of NK cells instead of using this drug in the late stages, concluding that these cells could be considered as therapeutic targets.

With respect to E2 modulation on the TAMs' function, there are not any reports that have evaluated its inhibition effect in clinical trials. We described before that E2 promotes alternative macrophage activation (72, 74). Interestingly, Hollmén et al. found that ER-positive and ER-negative tumors induced different macrophage phenotypes with different biological functions, morphology, and cytokine and chemokine secretion. In fact, alternatively activated macrophages present in triple negative breast cancer have a down-regulation in citrulline metabolism (80). From this concept, it would be interesting to study the effects of this hormone on citrulline metabolism, since it is known that nitric oxide synthase (iNOS) expression is enhanced by E2 action (166) and, simultaneously, this enzyme is associated with citrulline and arginine metabolism, determining the macrophages' activation phenotype in breast cancer (167). The number of neutrophils *in situ* in breast tumors is positively correlated with poor prognosis (102), so the modulation of their number could be interesting for breast cancer patients. In 2017, Dai et al. clearly demonstrated that estradiol treatment increased the number of neutrophils in the spleens of mice (105). An increased neutrophil number was also found in the complete blood of prostate cancer patients treated with estramustine (168), an antineoplastic agent with ER affinity (169). However, at present there are not any reports on neutrophil modulation in breast cancer tumors by ER inhibitors. On the other hand, it is known that in neutrophils, NETs formation is relevant for pathogen death, and a selective estrogen receptor modulator (SERM), raloxifene, inhibited NETs formation of human neutrophils, interfering with bacteria clearance after the treatment of the NET inducer phorbol 12-myristate 13-acetate (PMA) (170). This was opposite to the effect that was found with tamoxifen treatment (171). With respect to other immune populations, there are not any reports regarding their function modulation by ER inhibitors in breast cancer patients. In addition to the data described above, our workgroup reported that endocrine-disrupting compounds such as bisphenol A (BPA) have a significant effect on the modulation of ERα expression in T lymphocytes, macrophages, and NK cells of breast cancer tumors as well as in tumor growth. Impressively, a single administration of BPA in neonatal mice resulted in important changes in the presence of Tregs infiltrated into breast tumors in the adult stage (172). These facts provide new approaches to studying the effect of various compounds with estrogenic activity on the modulation of immune cells as well as in the selective inhibition of ER.

On the other hand, although different immunohistochemical studies as well as DNA sequencing data have given promising landscapes of infiltrating immune cells in this neoplasm for its therapy (13, 21), and despite the extraordinary efforts to reach a consensus on the study of the invasive population in breast cancer in daily histopathological practice (173, 174), different techniques such as flow cytometry must be applied in the clinic in order to guarantee precise studies. This is because it has been described that, according to the tumor area, the presence of infiltrating lymphocytes can vary (175). The above would allow offering personalized, predictive, and effective combined breast cancer treatments.

## Concluding Remarks and Future Directions

The main aim of this paper is to stand out the components of immune cells within the tumor microenvironment in different phenotypes of breast cancer, and the participation of E2 and its receptors in their function. As described above, E2 modifies the functions of different immune populations. Although the effects of this hormone were described in a particular way in each immune lineage, it is known that all of them are interconnected by cytokines, maintaining a dynamic interaction in the tumor microenvironment. Several reports have mentioned that immune infiltrating cells play a positive role in avoiding the progression of breast cancer and have a significant clinical impact on the response to treatment in a manner independent of the cancer phenotype (176, 177). However, little is known about their percentage and their grade of activation or anergy in different advanced clinic stages of this pathology, which might be modified due to the intratumoral E2 concentration. Based on the role that E2 and its signaling have in different populations of the immune system, we consider it important to evaluate or measure the intratumoral levels of this hormone and/or different compounds such as endocrine disruptors mainly in the advanced stages of this disease, which could be associated with their pro-anergic state. It has been documented that the concentrations of E2 as well as the enzyme that produces it (aromatase) are elevated inside the tumor (178, 179), affecting not only epithelial cell growth but also the immune cell effects. Taking into consideration the previous fact, we also regard the use of intratumoral therapy using ER inhibitors in the different types of breast cancer as an integral adjuvant approach for heightening both other therapies and immune response. The previous concept has taken on importance in cancer therapy; indeed, new studies on this topic are being done with different treatment schemes (180). Finally, the immune cells' function and their cytokines are key factors whose modulation should be studied, and they should also be considered as predictive markers and important therapy targets in different subtypes of breast cancer.

## Author Contributions

MS-M was in charge of all compilation of information, drafting of the manuscript, and participated in its conception. JM-M participated in the critical revision of the content of the manuscript and made a substantive intellectual contribution to drafting it. All authors read and approved the final manuscript.

### Conflict of Interest Statement

The authors declare that the research was conducted in the absence of any commercial or financial relationships that could be construed as a potential conflict of interest.

## Abbreviations

BPA: bisphenol A
cAMP: cyclic adenosine monophosphate
CAT: catalase
CG: cathepsin G
Da: daltons
DAG: diacilglycerol
DCs: dendritic cells
DNA: deoxyribonucleic acid
E2: estradiol
ERK: extracellular signal-regulated kinase
ER: estrogen receptor
EREs: estrogen response elements
G-CSF: granulocyte colony-stimulating factor
GFRs: growth factor receptors GPER1
GM-CSF: granulocyte macrophage colony stimulating factor
GPER-1: G protein-coupled estrogen receptor 1
GSH-Px: glutathione peroxidase
GSTP: glutathione S-transferase P
HER2: epidermal growth factor receptor type II
IFN-γ: interferon-gamma
Ig: immunoglobulin
IL: interleukin
iNOS: inducible nitric oxide synthase
IRF4: interferon regulatory transcription factor 4
LPS: lipopolysaccharide
MCs: mast cells
MCP-1: monocyte chemo-attractant protein 1
MMPs: metalloproteinases
NE: neutrophil elastase
NETs: neutrophil extracellular traps
NF-κB: nuclear factor-B
NK: natural killer cells
NO: nitric oxide
PAMPs: pathogen-associated molecule patterns
PI-9: proteinase inhibitor 9
PI3K/AKT: phosphoinositide 3-kinase
PR3: proteinase 3
PTGS2: prostaglandin-endoperoxide synthase
ROS: reactive oxygen species
SERM: selective estrogen receptor modulator
SOCS3: suppressor of cytokine signaling 3
SOD: superoxide dismutase
TAMs: tumor-associated macrophages
TET1: ten-eleven-translocation 5-methylcytosine dioxygenase
TCD4: helper T cells
TCD8: cytotoxic T cells
TGF-β: tumor growth factor beta
TLRs: Toll-like receptors
TNFα: tumor necrosis factor alpha
T regs: regulatory T cells
VEGF: vascular endothelial growth factor.
