# Erratum: Immune Tumor Microenvironment in Breast Cancer and the Participation of Estrogen and Its Receptors in Cancer Physiopathology

**DOI:** 10.3389/fimmu.2019.01868

**Published:** 2019-08-09

**Authors:** Frontiers Production Office

**Affiliations:** Frontiers Media SA, Lausanne, Switzerland

**Keywords:** immune infiltration, breast cancer, estrogen receptor, estrogen receptor inhibitors, tumor microenvironment

Due to an error in the editorial process, this article was not proofread before publication. The article has now been proofread and the necessary language-related corrections have been implemented. The previous version is available in the Supplementary Material of this Erratum. The publisher apologizes for this mistake. The original article has been updated.

