# Supplementary material for: Erratum: Immune Tumor Microenvironment in Breast Cancer and the Participation of Estrogen and Its Receptors in Cancer Physiopathology
Source: Front Immunol. 2019 Aug 9;10:1868. doi: 10.3389/fimmu.2019.01868 (PMC6696952; doi:10.3389/fimmu.2019.01868)
Supplement: Supplementary file 1 [file Data_Sheet_1.PDF]

frontiers in Immunology

REVIEW
published: 01 March 2019
doi: 10.3389/fimmu.2019.00348

# Immune Tumor Microenvironment in Breast Cancer and the Participation of Estrogens and Its Receptors Into Cancer Physiopathology

*Mariana Segovia-Mendoza and Jorge Morales-Montor**

*Departamento de Inmunología, Instituto de Investigaciones Biomédicas, Universidad Nacional Autónoma de México, Mexico City, Mexico*

Breast cancer is characterized by cellular and molecular heterogeneity. Several molecular events are involved in controlling malignant cell process. In this sense, the importance of studying multiple cell alterations in this pathology is overriding. A well-identified fact on immune response is that it can vary depend on sex. Steroid hormones and their receptors may regulate different functions and the responses of several subpopulations of the immune system. Few reports are focused on the function of estrogen receptors (ERs) on immune cells and their roles in different breast cancer subtypes. Thus, the aim of this review is to investigate the immune infiltrating tumor microenvironment and prognosis conferred by it in different breast cancer subtypes, discuss the current knowledge and point out the roles of estrogens and its receptors on the infiltrating immune cells, as well as to identify how different immune subsets are modulated after anti-hormonal treatments in breast cancer patients.

**Keywords: immune infiltration, breast cancer, estrogen receptor, estrogen receptor inhibitors, tumor microenvironment**

OPEN ACCESS

***Edited by:***
*Daniela F. Quail, McGill University, Canada*

***Reviewed by:***
*Luis De La Cruz-Merino, Hospital Universitario Virgen Macarena, Spain*
*Mallikarjun Bidarimath, Cornell University, United States*

****Correspondence:***
*Jorge Morales-Montor*
*jmontor66@biomedicas.unam.mx*

***Specialty section:***
*This article was submitted to Cancer Immunity and Immunotherapy, a section of the journal Frontiers in Immunology*

***Received:*** *30 November 2018*
***Accepted:*** *11 February 2019*
***Published:*** *01 March 2019*

***Citation:***
*Segovia-Mendoza M and Morales-Montor J (2019) Immune Tumor Microenvironment in Breast Cancer and the Participation of Estrogens and Its Receptors Into Cancer Physiopathology. Front. Immunol. 10:348. doi: 10.3389/fimmu.2019.00348*

## INTRODUCTION

### Breast Cancer and Infiltrated Immune Cells Microenvironment

Breast cancer is the most frequently diagnosed malignancy in women worldwide, it does represents the second most common cause of cancer deaths (1). Epidemiological studies have indicated that steroid sexual hormones play important roles in the initiation and progression of breast cancer. Besides, other risk factors are associated with this disease such as diet, ethnic differences, age, early menarche, not bearing children, first pregnancy over the age of 30, obesity, genetic mutations, oral contraceptives exposure, consumption of alcohol or cigarettes, and environmental contaminants, among others. It is estimated that more than one million women are diagnosed with breast cancer every year, and more than 410,000 will die from the disease (2, 3). The above indicates that breast cancer represents an important worldwide health problem.

On the other hand, breast cancer is a heterogeneous disease, which is traditionally classified in three phenotypes: luminal (estrogen receptor (ER) positive), human epidermal growth factor receptor type 2 (HER2) positive and triple negative (ER- negative/HER2- negative) (4). Moreover, breast cancer is characterized by high inflammatory microenvironment, which is supported by the infiltrating immune cells, cytokines, and growth factors (5, 6). In addition, immune infiltration of breast tumors has been related with clinical outcome trough of the modulation of treatment response. Breast tumors with immune infiltration are associated with different patterns based

on ER presence, however, a common bad immune feature is that regulatory T cells (T regs) are associated with poor prognosis in both ER-positive and ER- negative breast tumors, conferring an immunosuppressive environment (7, 8). Such feature it is a characteristic that highlights the importance of immune tumor microenvironment in breast cancer.

With respect to other infiltrating immune cells in breast cancer phenotypes, a strong proportion of natural killer cells (NK) and neutrophils have been found in ER-positive breast tumors, while cytotoxic T cells ($TCD8^+$), naïve and memory T cells ($TCD4^+$) are found in less proportion. Interestingly, eosinophils and monocytes are associated with a good response after chemotherapy; also, B lymphocytes are associated with good prognosis in this phenotype. Recently, as a part of good prognosis in this type of tumors, activated mast cells have been correlated with good prognosis (9). However, the presence of this population is still controversial (10). Moreover, in this phenotype, tumor-associated macrophages (TAMs) 1 and 2, and T regs lymphocytes showed poor prognosis due to their inflammatory, immunosuppressive and pro-tumorigenic role (11–14). In ER-negative breast tumors, the major component of immune infiltration cells are T regs, TAMs2 and activated mast cells, which are also associated with bad prognostic. On the counterpart, $TCD4^+$, $TCD8^+$, B lymphocytes, and dendritic cells (DCs) are related with better prognosis, but they are found in less proportion and they can be associated with the favorable response to neoadjuvant chemotherapy (7, 13, 15–21). With respect to HER2 positive breast cancer type, there are not many reports about the infiltrating immune mass. However, it is mainly represented by DCs, mast cells, γδ T lymphocytes, T regs, and neutrophils, interestingly all of them confer poor prognosis, disease relapse and metastasis in this phenotype (**Figure 1**) (13, 22, 23).

This intra-tumoral immune pattern establishes a complex relationship among the heterogeneity of immune infiltrating cells, the tumor phenotype and the treatment response in breast cancer.

**Abbreviations:** BPA, bisphenol A; cAMP, cyclic adenosine monophosphate; CAT, catalase; CG, cathepsin G; Da, daltons; DAG, diacilglycerol; DCs, dendritic cells; DNA, deoxyribonucleic acid; E2, estradiol; ERK, extracellular signal-regulated kinase; ER, estrogen receptor; EREs, estrogen response elements; G-CSF, granulocyte colony-stimulating factor; GFRs, growth factor receptors GPER1; GM-CSF, granulocyte macrophage colony stimulating factor; GPER-1, G protein-coupled estrogen receptor 1; GSH-Px, glutathione peroxidase; GSTP, glutathione S-transferase P; HER2, epidermal growth factor receptor type II; IFN-γ, interferon-gamma; Ig, immunoglobulin; IL, interleukin; iNOS, inducible nitric oxide synthase; IRF4, interferon regulatory transcription factor 4; LPS, lipopolysaccharide; MCs, mast cells; MCP-1, monocyte chemo-attractant protein 1; MMPs, metalloproteinases; NE, neutrophil elastase; NETs, neutrophil extracellular traps; NF-κB, nuclear factor-B; NK, natural killer cells; NO, nitric oxide; PAMPs, pathogen-associated molecule patterns; PI-9, proteinase inhibitor 9; PI3K/AKT, phosphoinositide 3-kinase; PR3, proteinase 3; PTGS2, prostaglandin-endoperoxide synthase; ROS, reactive oxygen species; SERM, selective estrogen receptor modulator; SOCS3, suppressor of cytokine signaling 3; SOD, superoxide dismutase; TAMs, tumor-associated macrophages; TET1, ten-eleven-translocation 5-methylcytosine dioxygenase; TCD4, helper T cells; TCD8, cytotoxic T cells; TGF-β, tumor growth factor beta; TLRs, Toll-like receptors; TNFα, tumor necrosis factor alpha; T regs, regulatory T cells; VEGF, vascular endothelial growth factor.

## ESTROGEN SIGNALING AND ESTROGEN EFFECTS IN BREAST CANCER CELLS

Estradiol (17β-estra-1,3,5(10)-triene-3,17-diol) E2 is a steroid hormone produced by theca and a granulosa cell in the ovaries. E2 regulates several physiological and pathological processes including cancer. Classical or genomic E2 signaling is mainly mediated by two isotypes of the receptor: ERα and ERβ, both of which are nuclear transcription factors that bind to their specific ligand or several estrogens in general; and subsequently they form homo or heterodimers that bind to estrogen response elements (EREs) contained in the promoter region of specific genes in order to activate or suppress their expression; these actions are mediated by the distinct co-activators or co-repressors recruitment or through the interaction with other transcription factors (**Figure 2**) (24). E2 actions are also mediated by other non-classical pathways, known as ligand-independent ERα signaling, by a membrane-anchored receptor called G protein-coupled estrogen receptor 1 (GPER1), in which target gene transcription occurs through second messengers and several transcription factors. Thus, GPER1 mediates the increase of different second messengers such as cyclic adenosine monophosphate (cAMP) and diacilglycerol (DAG) levels, mobilization of intracellular calcium ($Ca^{2+}$) and the activation of extracellular signal-regulated kinase (ERK)1/2 and the phosphoinositide 3-kinase (PI3K/AKT) pathways by the trans-activation of the different growth factor receptors (GFRs). Moreover, the activation of GPER1 can induce the release of several growth factor ligands such as heregulin, which results in a direct activation of GFRs, **Figure 2** (25–28). It is important to mention that different antagonists or ER inhibitors such as ICI 182,780 and tamoxifen, can mimic the effects of estradiol and induce GPER1 activation.

In breast cancer, $E_2$ can act in different ways. For instance, in immortal cell lines of breast cancer, E2 via ERα signaling shows to stimulate proliferation, while ERβ activation inhibited cell proliferation and promoted apoptosis (29, 30). Interestingly, estrogens can also undergo several metabolic processes and its metabolites exert genotoxic effects, which contribute to the development of breast cancer through adduct DNA formation (31–33). Many reports about the effects of E2 in breast cancer cells, have reported the transcriptional modulation of different genes that are affected; among which are: proliferation regulators, growth factors, cell cycle, and apoptotic modulators (29, 34, 35).

Importantly, both, classic and membrane ERs have been implicated in several effects of immunity and autoimmunity (36, 37). It is known that the immune system shows remarkable sex differential responses: thus, this fact potentially suggests that sex hormones such as estrogens address these events. Therefore, many reports mention that women respond more aggressively to self-antigens, being more susceptible to autoimmune diseases through of the activation of ER signaling (38). In general, ERs participate in many immune system functions, ERα has been related in spleen, and thymus function, while ERβ is important in the bone marrow functions (24). Both types of ERs are expressed on innate and adaptive immune system cells, indicating an

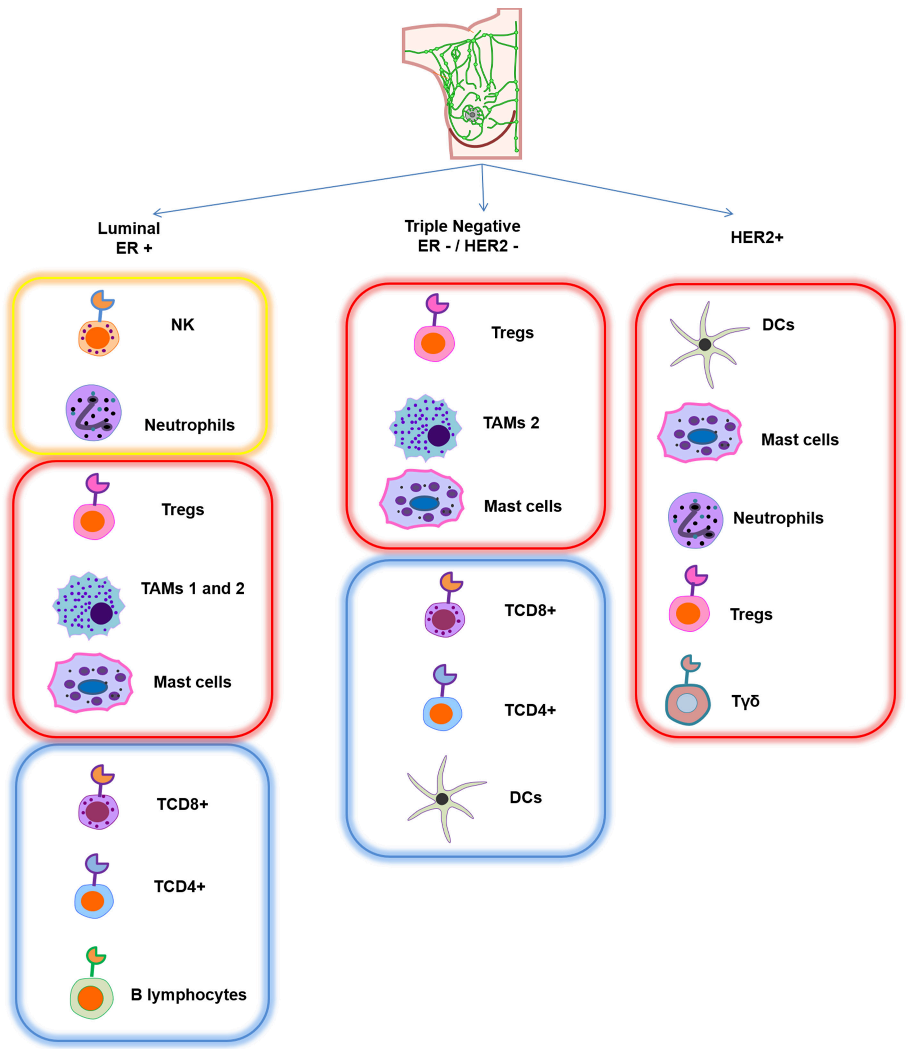


**FIGURE 1 |** Schematic representation of the main infiltrating immune cell pattern in different breast cancer subtypes. Each subtype has different composition of immune cells, yellow frame represents strong presence of specific immune cells that confer good prognosis, red frame indicates that this infiltrating signature is associated with poor prognosis and blue frame corresponds to less proportion of immune cells that are also related with good prognosis.

important role of this hormone and its receptors signaling in the correct immune performance (39).

We describe below the modulation of the most common tumor infiltrating immune cells by estradiol action upon binding to its receptors in these immune cells of the tumor microenvironment.

## ESTROGEN EFFECTS ON IMMUNE SYSTEM CELLS

### ER in Dendritic Cells (DCs)

DCs are involved in several processes such as immune tolerance, autoimmunity, stimulation, and differentiation of naïve T cells. They are considered as a potent antigen presenting cells (APC), they are mainly activated by stress or damage signs from pathogens that are recognize mainly by Toll-like receptors (TLRs). Following their stimulation via TLRs, DCs secrete pro-inflammatory cytokines to stimulate T lymphocytes and initiate the innate immune response. In this sense, ER participates in the favoring of DCs function. These cells contain the presence of ERs, when its ligand binds to ERs in these cells; it can trigger migration and activation process. In addition, in mouse *in vitro* models of DCs, estrogen can induce differentiation, survival, and increase the expression of co-stimulatory molecules (39). It has been reported that pre-treatment of E2 in co-cultures of mature DCs with T cells resulted in the stimulation of T cells proliferation (40). Besides, E2 up- regulates the expression and secretion of different pro-inflammatory cytokines and chemokines such as tumor necrosis factor alpha (TNFα), interleukin (IL)-6, CXCL-8 (IL-8), and monocyte chemo-attractant protein 1 (MCP-1) (40). This concept can be directly related to the improvement of DCs

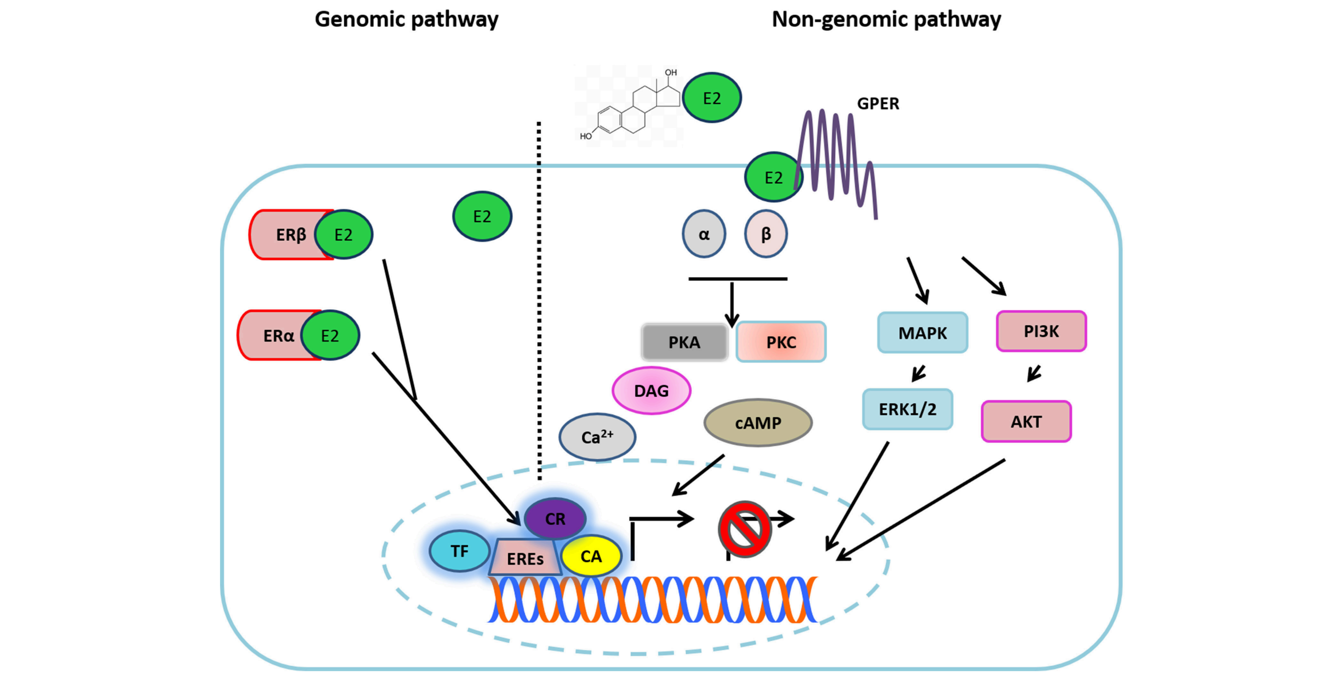


**FIGURE 2 |** Estradiol signaling. Estradiol (E2) can bound to its different receptors for activating the genomic pathway or the non-genomic pathways. In the first one, E2 binding to ERα and ERβ, each complex is directed to the nucleus where it joins to EREs in the DNA recruiting different transcription factors (TF), co-activators (CA), or co-repressors (CR) in order to activate or suppress the transcription of target genes. In the non-genomic pathway, E2 binds to GPR30 triggering the activation of G proteins, the above turns out in the increase of different second messengers, (cAMP, $Ca^{2+}$, DAG) Besides, the E2 through the non-genomic pathway can activate different growth factor receptors (GFR) activity which results in the activation of different downstream signaling pathways (MAPK and PI3K) and in the release of different ligands of GFRs.

capability to mediate presentation of self and foreign antigens, and maybe because of this, the immune system response against to the tumors is better in early stages of the disease. Nevertheless, the presentation process is disrupted by E2, since after hormone exposure, production of INF-γ, and IL-2 is decremented in mature DCs (41). Suggesting that the E2 effects in DCs depends on their maturation stage. Thus, it would be interesting to determine the degree and phenotype of DCs maturation in tumors. In addition, differentiation of functional DCs from bone marrow can be also modulated by this hormone, since it favors their migration to lymph nodes, effect that was reverted with the use of specific ERα antagonist (ICI 182,780) (42–44). Supporting this notion, E2 induces myeloid DCs differentiation through the activation of two inflammatory-related proteins, the interferon regulatory transcription factor 4 (IRF4) and the participation of granulocyte macrophage colony stimulating factor (GM-CSF). Interestingly, it was reported that the exacerbated activation of these two factors by E2 in some point can lead a DCs tolerogenic phenotype (45). The association of ERα with other proteins such as thiolase, glutathione S-transferase P (GSTP) is also linked with DCs differentiation. In addition to this, metabolic function, several growth factors, and accessory proteins in bone marrow derived mice DCs are also affected. On contrary, the absence of GSTP enhanced DCs metabolism, their proliferative and differentiation rates and their effector functions (46). It is important to note that not only E2 has effects in DCs, an estradiol metabolite, estriol also generated tolerogenic DCs in an *in vivo* model that protect against autoimmunity (47). The above points out to monitor the effects of ER inhibitors on different immune cell functions, favoring not only the inhibition of cancer cells but also the migration of the immune ones to lymph organs or avoid their anergic phenotype.

## ER in Macrophages (Mø)

Macrophages are a fundamental part of the innate defense mechanisms against foreign pathogens, which can promote specific immunity by inducing T cell recruitment and activation. Their role is essential for triggering the adaptive immune response. Macrophages collaborate with T and B cells based on the release of cytokines, chemokines, reactive radicals, among other proteins. Despite this fact, their presence within the tumor microenvironment has been associated with enhanced tumor progression and promotion of cancer cell growth, angiogenesis, and immunosuppression (11, 48).

Several articles have reported the presence of ER in monocytes, macrophages precursor cells (49, 50), the expression of this hormone receptor varies between stages of differentiation, monocytes expresses ERβ while macrophages express ERα (51), although recently, both receptors have been found in macrophages (52). E2 treatment has shown to modulate different macrophages actions and their metabolism, for example, it is well know that production of nitric oxide (NO) into the

macrophages allow them to exert antimicrobial and antitumor actions (53), related to this concept, hormone treatment stimulated the NO release in human peripheral monocytes and in murine macrophage cell line via GPER activation coupled with intracellular calcium influx (54, 55). In line with that, the stimulation with LPS in isolated peritoneal macrophages coming from young female rats resulted in an elevated NO release, this effect was not observed in macrophages derived from the middle-aged animals, where circulating E2 levels were diminished (56). Moreover, macrophages produce and use arachidonic acid and its different metabolites for the recognition of pathogens and to enhance or suppress inflammatory response (57). E2 has shown to modulate the lipid metabolism of macrophages, since it elicited an increase of arachidonic acid release and prostaglandin E2 production (a derivative of arachidonic acid) in a human monocytic cell line (58). In addition, the phagocytic activity of macrophages is performed in part by reactive oxygen species (ROS), which cause DNA or cell membrane damage, the interplay between intracellular ROS and antioxidant enzymes such as superoxide dismutase (SOD), catalase (CAT), and glutathione peroxidase (GSH-Px) is important in the macrophage phagocytic function, activation, differentiation and recruitment process (59). In this context, it has been reported that E2 administration in rats modulated the CAT activity in an *ex vivo* macrophages (60). As part of the bacterial killing mechanism by macrophages induced by LPS are the activation of metalloproteinases (MMPs); especially the gene expression of MMP-9 was dramatically reduced after E2 treatment in rat cell lines of macrophagic origin, this effect was blocked with ICI 182,720 treatment (61). This hormone also modulates the macrophage survival, this effect was reported in an *in vitro* culture of human macrophages where E2 treatment induced the anti-apoptotic protein Bcl-2, action mediated by the modulation of intracellular $Ca^{+2}$ concentration, the activation of protein kinase C and ERK phosphorylation (62, 63). Furthermore, macrophages can recognize distinct pathogen-associated molecule patterns (PAMPs) which contributes to activate several signaling cascade and diverse cytokines and chemokines (64). E2 via ERα reduced the gene and protein expression of the pro-inflammatory IL-8 in monocytes previously challenged by LPS (65). The modulation of this chemokine impacts not only in the macrophages function but also in the neutrophils recruitment to inflammation sites, mediating pathogen clearance (66). E2 also can modulate other functional macrophage cytokines; its treatment decreased the IL-6, TNF-α, IL-1β expression in whole blood cultures derived by healthy postmenopausal women, in bone marrow cell cultures and in an *ex vivo* rat macrophages (56, 67–70). The modulation of these cytokines was confirmed to be E2-dependent effect, according to the opposite event found in these cells when they were treated with ICI 182,780 (69). Similar result of E2 treatment related to the decreased expression of TNF-α gene was reported in a ER-positive murine monocytic cell line through of the down-regulation of Jun NH(2)-terminal kinase activity with the consequent decrease on AP-1 transcription factor, affecting the TNF-α transcription (71). In addition, E2 modulates the macrophages activation (72), which is mainly classified in two categories; classical activation (macrophages kill microbes and act as an anti-tumor effector cells) which is promoted by IFN-γ and TNF-α; TH2 related cytokines or alternative activation (macrophages lay down extracellular matrix components to promote wound healing, angiogenesis and sustain tumor progression). This type of macrophage activation is promoted by TH1 cytokines, being a IL-4/IL-13 dependent mechanism (73). The effect of this hormone in macrophages activation was clearly observed in a murine wound healing model in ovariectomized mice. In this sense, macrophages coming from ovariectomized animals shown preferentially a classical activation, in addition, the gene expression of two alternative activation macrophages markers (Fizz1 and Ym1) were reduced, and also the ovariectomized mice presented a reduction in both, macrophage numbers in the wound area and the inflammatory environment through the reduction of monocytes-associated TNF-α secretion as compared with the intact group. On contrast, E2 supplementation in ovariectomized mice restored the expression of both markers, directing into an alternative macrophage activation, wound repair, remodeling and angiogenesis (72). Furthermore, the alternative macrophages activation promoted by E2 has been documented in other assays. With respect to this notion, the gene expression of arginase 1, another established alternative activation macrophage marker, was up-regulated with an ERα agonist treatment in an *in vitro* culture of bone marrow-derived macrophages (74). This work also evaluated the role of E2 in wildtype or in an ERα and inflammatory gene deletion mice (LysM-ERα) subjected to incisional wounds with a subsequent exogenous E2 replacement. Of note, in the hormone absence, healing was delayed (74) as has been previously reported in ovariectomized wildtype mice model (72). However, the hormone treatment revealed increased recovery in healing response whereas in ERα knockout mice resulted in a marked healing delay, the above mentioned highlight the role of estradiol-ERα action in the induction of alternative macrophage activation (74). Besides, the role of E2 to favor alternative macrophage activation was corroborated in an *in vitro* and *ex vivo* study in human blood-derived macrophages. In fact, classical lipopolysaccharide (LPS)/IFN-γ stimulus on un-polarized macrophages induced the down-regulation of two markers of alternative activation (CD163 and CD206), these effects were avoided with its treatment through of the modulation of NFκB transcription factor (75). Interestingly, a lot of evidence support that macrophages, especially, alternatively activated macrophages shape immune tumor infiltration and have influence in high vascular grade related with metastasis (76–79). In this sense, breast cancer phenotype also can regulate the type of infiltrating macrophages phenotype (80). Current evidence suggests that this population of macrophages regulate at the same time the ERα expression in an epigenetic manner, through the modulation of a DNA hydroxymethylation marker, ten-eleven-translocation 5-methylcytosine dioxygenase (TET1). The above was demonstrated in co-cultures of endometrial cancer cells with alternatively activated macrophages, the result shown that alternatively activated macrophages enhanced both E2-driven endometrial cancer cell proliferation and upregulation in ERα expression, mechanism dependent of IL-17A expression (81). The above highlights the importance

of the interplay among sex steroids, immune system and tumor progression.

## ER in Mast Cells

Mast cells (MCs) are tissue-resident immune cells that form part of the innate immune system. They are commonly associated with allergic reactions and parasitic infections. These cells are characterized by the presence of granules loaded with different inflammatory mediators that they release depending on the time and the type of stimulus (82). Additionally, secretion of serine proteases such as tryptase or chymase define what phenotype of mast cell will be activated, it means that mucosal mast cells produce tryptase and connective tissue mast cells secrete tryptase, chymase and carboxypeptidases (83). These enzymes in conjunction with the release of IL-8, tumor growth factor beta (TGF-β), and TNF-α have been related with angiogenesis trough vascular endothelial growth factor (VEGF) and MMPs modulation in different breast cancer phenotypes (9, 84). Mast cells can be activated by the directly recognition of pathogen-associated molecular patterns (PAMPs) or by immunoglobulins and immunoglobulin E receptor (FcϵRI) interaction; both cases result in the release of different molecules from their granules, recruiting different immune cells.

On the other hand, several studies have reported the presence of ERα but not ERβ in mast cells, however, recently, it was described that these cells have the presence of both nuclear receptors (85–88). In this sense, treatment of E2 or an endocrine disrupting compound such as bisphenol A have demonstrated to induce the release of histamine (an important biomolecule involved in allergic reactions) from rat mast cells in a concentration-dependent manner (89). Of note, the histamine release is also important in breast cancer promotion since this protein or its receptors (H3R and H4R) have been related with the induction of breast cancer cell proliferation and migration. Importantly, these molecules have been identified to a greater extended in breast tumor samples as compared with non-tumor samples (90) the above suggests that the inhibition of this molecule could result in an interesting target in this disease. E2 has an important role for inducing the release of asthma mediators such as leukotriene and β-hexosaminidase in a rat mast cell line. The release of β-hexosaminidase also has been described in both, human mast cell line and in a primary culture (non-transformed) of mast cells. This action was blocked with the addition of tamoxifen or ICI 182,780; demonstrating that ERα is responsible of these actions (89, 91, 92). In relation to breast cancer progression, tryptase release from mast cells has been closely related with increased number of carcinoma-associated fibroblasts in breast tumor samples, favoring the tissue remodeling and angiogenesis (93). Related with this, E2 up-regulates the tryptase secretion in the human mast cell line HMC-1 (88), assuming that it induces the degranulation of these cells. In addition, E2 in an *ex vivo* model induces the expression of two chemokine receptors (CCR4 and CCR5) which are implicated in the migration of periphery mast cells to the uterus (88, 94). The above highlights the effects of E2 in mast cell function with the purpose of favoring breast cancer progression. On the other hand, there are few reports with respect of E2 function by non-genomic pathway in mast cells. In this regard, it has been shown that estradiol induces the release of intracellular calcium, which is important for degranulation and leukotriene synthesis in mast cells (95). Recently, the role of mast cells in breast cancer has been largely studied (10), however, many of their functions and components in their granules in relation with breast cancer progression are still little addressed this makes them an important population for their study in the cancer microenvironment.

## ER in Neutrophils

Neutrophils constitute the first line of host defense; they are other fundamental pathogen-fighting immune cells. Neutrophils can be recruited to infection sites and eliminate microbes by classical phagocytosis or degranulation, also they produce ROS, release antimicrobial peptides or expel their nuclear content in order to form the neutrophil extracellular traps (NETs) (96, 97). Neutrophils collaborate with other immune cells such as macrophages or DCs; they secrete many chemokines and cytokines that regulate the immune response (98). It has been described that neutrophils as well as other immune cells present both nuclear receptors (99). In this regard, E2 through ERα binding has been shown to regulate neutrophil survival, function and number. E2 exposure delayed the apoptosis in human neutrophils this effect was correlated by a significant decrease in active caspase 3 protein expression and it was reverted by ICI 182,780 treatment (100). This represents a possible explanation of the sexual dimorphism being that neutrophil number differs between male and women (101). As a part of E2 in neutrophils functions, it enhanced the NO production and the nitric oxide synthase in human neutrophils (102, 103). Additionally, neutrophils secrete several serine proteases (NSPs) including neutrophil elastase (NE), proteinase 3 (PR3), and cathepsin G (CG), which are essential for neutrophils infectious agents elimination and the modulation of inflammation (104). Neutrophils derived from splenocytes of mice administered with E2 shown a NE, PR3, and CG increment in gene and protein expression as compared with placebo treated mice. Moreover, E2 administration in these mice increased the number of them in different lymphoid tissues (splenocytes, peripheral blood, and bone marrow) and the gene and protein expression of myeloperosidase, a major component of neutrophils granules (105). E2 via ERα modulated the inflammation and the actions mentioned above were related with an autoimmune disease; since an increase of neutrophil number and NSPs were found in mice with lupus (105). Moreover, G1-GPER1activation also participates in the neutrophil polarization (analogous concept of macrophage activation) (106), promoting the gene expression of pro-inflammatory phenotype (N2) and their life span, actions mediated by the activation of cAMP/PKA/CREB, MAPK and p38 signaling pathways (107). This work also shown that IL-1β, IL-8, the prostaglandin-endoperoxide synthase (PTGS2), the suppressor of cytokine signaling 3 (SOCS3), and the granulocyte colony-stimulating factor (G-CSF) gene expression was enhanced after the stimulation of G1-GPER1 in a dose dependent manner, also the IL-8 release was significantly increased as compared with non-treated human neutrophils

and with neutrophils stimulated with LPS. Furthermore, this hormone-receptor interaction up-regulated the surface expression of two markers of neutrophil activation (CD11b and CD62L) (107). Supporting the fact that G1-GPER1 interaction is responsible for the IL-8 neutrophil release. Other work proved that 17β-estradiol-ERα did not induced the release of this chemokine, in fact, the estradiol treatment has the opposite effect in the release of this chemokine in human neutrophils pre-stimulated with LPS (108). In addition, this classical activation may participate in the attenuation of the neutrophil activation; E2 reduced the shedding of a surface adhesion neutrophil molecule (CD62L or selectin) (108), that is normally implicated on diapedesis at sites of tissue injury and inflammation (109). Also, E2 treatment blocked the neutrophil chemotaxis promoted by IL-8; the generation of superoxide anion by neutrophils was diminished with this hormone treatment (108, 110, 111), affecting their host defense function (112).

It is well-known that a certain type of breast cancer is dependent of E2 action; coupled with this notion, this hormone can promote inflammation through the induction of neutrophil infiltration and the expression of pro-tumoral cytokines/chemokines, and tissue-remodeling enzymes in mammary neutrophils (113). In a mammary involution mice model, E2 administration induced mammary neutrophil infiltration and neutrophil pro-tumoral activity signature, since at least 10 inflammatory genes were up-regulated in mammary resident cells, interestingly; neutrophil depletion reversed these inflammatory genes expression pattern. Moreover, in this mammary involution mice model, the mice were administrated with E2 and injected with a triple negative breast cell line (4T1), again, the hormone treatment induced mammary neutrophil infiltration, however, neutrophil depletion, with an specific antibody, resulted in the markedly abolition of estrogen-induced mammary tumor growth (113). The mammary neutrophil recruitment induced by this hormone was observed in other *in vitro* and *in vivo* breast cancer study, in which it promoted N2-neutrophils polarization, correlated with the integrin LFA-1 and TGF-β overexpression, intra- and extravasation and trans endothelial breast cancer cell migration and with major breast tumor growth, last effect was reverted by ICI 182,780 treatment. In fact, E2 treatment became a non-metastasic breast cancer cell line into metastatic associated with neutrophils presence (114). The previous facts give to mammary neutrophils presence and activity a significant implication in cancer progression whose are importantly regulated by E2.

## ER in NK Cells

NK cells are central components of the innate immunity and they participate in preventing and controlling infections and tumor growth and metastasis (115). Usually, in tumors there is a downregulation of self-ligands and expression of stress-induced ligands which can be recognized by NK cells (116). Their activation also leads to secretion of stimulatory cytokines and chemokines such as IFN-γ, TNF-α, GM-CSF, MIP1-α, and RANTES, which participate in the stimulation of the adaptive immune system. Moreover, their biological importance lies on their ability to exert its cellular cytolytic effect through of the liberation of granzymes and perforin (117).

Since the 90s, it is known that E2 causes the reduction in NK cell cytotoxic activity in mice models in a dose-dependent manner (118, 119). This data was confirmed when the hormone was administrated in postmenopausal and premenopausal women, resulting in a reduction in NK cell activity (120). In fact, the use of oral contraceptives, that bind to sex steroids receptors, have been related with changes in NK cytotoxic activity and with an increase on infections (121). Interestingly, the suppressive effect of E2 on the NK cells was attributed to the enhancement of metastasis in a fibrosarcoma and melanoma cell model, where, immunosuppressed mice treated with this hormone also exhibited deficient NK cell activity and increased susceptibility to develop metastasis of allogeneic tumor cells (122). Besides, synthetic non-steroidal estrogens such as diethylstilbestrol shown the same effects about inhibitory NK cells activity and the mice susceptibility to generate tumors derived from this NK cellular inhibition. Of note, the NK inhibitory activity was dramatically affected only with one neonatal administration of diethylstilbestrol into the mice (123). On the other hand, it has described that E2 can induce or suppress mice NK cells activity being actions dependent on time, at short times it acts in a stimulating way and at long times, it suppresses NK cells activity (124). Estrogen can also inhibit NK cell-mediated apoptosis due to this hormone induced a granzyme inhibitor named as proteinase inhibitor 9 (PI-9) (125). Nowadays, there are few reports that evaluate the effects of E2 in NK cells. However, it is known that the reduction of their activityis related with promotion of tumor growth (126), therefore, NK cells might be considered as a target for immune therapies in order to avoid the estrogen-mediated increase breast tumor incidence.

## ER in B Cells

B lymphocytes are part of the adaptive immune system that is specialized in the antibody production, which is part of the humoral immunity (127). It has been described that B lymphocytes have the expression of both nuclear ERs in all B cells subsets (39, 128). In this sense, E2 has stimulatory effects on B differentiated lymphocytes derived from human PBMCs. It increased the immunoglobulin (Ig)G and IgM production in a dose dependent manner, this effect was enhanced by the addition of IL-10, an anti-inflammatory cytokine, to B cells previously treated with E2 (129), the above becomes relevant in an autoimmune context. The stimulatory effect of E2 on the antibody titers has been observed since the 80s on *in vitro* studies and in the serum of rats administered with this hormone, where an increase in IgM antibodies was reported (130, 131). Of note, it has been reported that IgMs have a direct cytotoxic effect on transformed cells, trough the activation of the complement pathway (132, 133). This is relevant since the increases on IgMs levels due to E2 exposure are important for breast cancer suppression. Besides, they also might serve as diagnostic indicators of the phenotype or stage of this pathology, due to the fact that they are well-correlated with the clinical score and disease spread of breast cancer patients (134); however, more studies are necessary to affirm this fact. Added to that, E2 through

ERα pathway also impacts on the activation and survival of B cells through the modulation of several genes. These effects were observed in splenic B cells derived from ovariectomized mice or not, administered with it. Interestingly, these results were reverted in mice treated with ICI 182,780 (128). Regarding to effects of GPR30 on B lymphocytes, some reports have mentioned that different chemokines can activate it triggering different roles of B subsets such as migration, chemotaxis, proliferation, and apoptosis, among others. In fact, this receptor has been correlated with different B cells malignancies such as leukemia and lymphomas (135, 136). Nevertheless, more information or mechanisms of action related with this topic would be interesting in relation to the breast cancer pathogenesis.

## ER in TCD4$^+$ and TCD8$^+$ Cells

Lymphocytes have important roles in immune protection; traditionally these cells are divided in two subtypes, TCD4$^+$ and TCD8$^+$. The first subtype can help to B cells to produce antibodies, in order to induce the immune response through of the activation of macrophages and the recruitment of different immune cells to specific sites with inflammation. The second type is important for the defense against to cellular pathogens, among other functions. These immune populations can contribute to attenuate inflammation, production of antibodies and protection of pathogens (137). Based on the different cytokine secretion profile, TCD4$^+$ are divided in different subsets, for instance, T helper (Th)1 and Th2. Th1 are characterized by secrete INF-γ, IL-2, IL-12, and TNFα, cytokines that stimulate macrophages functions, cellular response; while Th2 cells secrete IL-4, IL-5, IL-6, and IL-10, important cytokines for the B cell antibody production, humoral response.

It has been described that E2 affects the size, maturation and development of T cells, process known as thymic atrophy (37, 138), this effect is mainly caused by ERα signaling (139). Moreover, it also can influence the expression of CD4$^+$/CD8$^+$ T cells phenotype and their subsets functions (140), and it also contributes to the development of other T cells subtypes from the lymph nodes, such as Th17 cells (141). Interestingly, the proliferation and generation of active T cells are governed by different metabolic glycolytic demands (142). In this sense, the orphan nuclear hormone receptor, estrogen-related receptor-α (ERRα) is a key regulator that supports T cell functions, since the inhibition of ERRα decreased several glycolytic genes implicated in the inflammatory cytokine production and T cell proliferation in an *in vitro* and in an experimental autoimmune encephalomyelitis mice model, similar effect found in ERRα deficient T cells (143). Several studies have demonstrated that E2 modulates IFNγ-secretion of Th1 cells in both human and mice cells, which is potentially mediated by direct interaction of ER with its EREs in the promoter region of the IFNγ gene (38, 144, 145). This cytokine has a pivotal role against to intracellular infections, autoimmune and inflammatory disorders. Furthermore, E2 inhibits the production of Th1 pro-inflammatory cytokines such as IL-2, IL-12, IFN-γ, and TNFα (146). In accordance with this notion, the declination of ovarian function related to menopause state in women and reduction in this hormone production has been related with an increment in pro-inflammatory cytokine production (147). In line with that, Th1 related cytokine levels such as IL-2, IFN-γ were augmented in postmenopausal women and the hormone replacement therapy in these population caused a significant decreased of these cytokines (148). On the other hand, the effects of E2 in Th2 cells are related to the increment of anti-inflammatory cytokines such as IL-10, IL-4, and TGF-β (146, 149). In addition, the IL-4 increment has been correlated with the increase of an essential Th2 transcription factor (GATA-3) (150). Interestingly, E2 administration in a mammary involution mice model diminished CD4$^+$ and CD8$^+$ T cells in mammary tissue, highlighting the effects of this hormone on the function of these immune cells type (113).

## ER in Regulatory T Cells (Tregs)

Tregs are involved in self-tolerance, suppression of immune cell functions, down-regulation of self-reactive lymphocytes action, and prevention of transplant rejection through activation of a lineage-specific transcription factor that governs Treg development, differentiation, maintenance, and function, forkhead/winged helix transcription factor (FoxP3) (151). As part of the T regs immunosuppressive T cell inflammatory activity are the IL-10 secretion and the induction of programmed cell death 1 receptor (PD-1) (137, 152). In breast cancer, these cells are related with a high rate of relapse and with favoring the tumor microenvironment (7, 16).

E2 *in vitro* and *in vivo* mouse model has shown to induce the gene expression of FoxP3 and IL-10. These effects were reverted with the treatment ICI 182,780 (153, 154). It also modulates the Tregs inhibitory capacity, since estradiol treatment increased intracellular PD-1 levels in Tregs cells coming from splenocytes of wild type mice, opposite effect seen in ER knockout mice (155). E2 treatment also has shown to promote the proliferation and the number of human Treg cells. In addition, it favors the change to CD4$^+$, CD25$^-$ cells to CD4$^+$, CD25$^+$ Tregs phenotype (156). Interestingly, a recent work demonstrates that infiltrating Tregs cells derived from human cervical cancer contain elevated levels of estrogen. Also, E2 through ERα signaling binds in the EREs of the Tregs FoxP3 promoter, in this way, a loop is formed and leads the activation of FoxP3 activity (157). As well as in other works, ICI 182,780 treatment reverted the E2 effects in Tregs cells and resulted in the ablation of FoxP3 protein expression and a decrease of TGF-β secretion (157). Another study supports the notion that in addition of ERα signaling, GPER with the estrogenic small molecule (G-1) is critical for the expansion of Tregs and the induction of Foxp3 protein in *ex vivo* cultures of purified TCD4$^+$ mice cells. In addition, G-1-GPER activation was able to maintain the Tregs phenotype and to induce the expression of two proteins implicated in control of immune homeostasis, PD-1, and cytotoxic T lymphocyte associated protein 4 (CTLA-4), in the presence of Th17 cytokine inflammatory polarization conditions (158). It has been described that Tregs secrete immunomodulatory cytokines such as TGF-β and IL-10 (159). This cytokine secretion pattern was favored with E2 treatment in Tregs cells isolated from peripheral blood mononuclear cells (PBMCs) of healthy women (160). The previous data highlight

the fundamental role of estradiol-ERα / G1-GPER pathway in Tregs cell physiology.

Estrogens pathways on immune cells studied in basal condition described above are illustrate in **Figure 3**, which highlights that few reports have evaluated Tregs, macrophages, neutrophils, and mast cells effects mediated by non-genomic pathways. It also aims to represents how immune infiltration is found in breast cancer. In addition, **Table 1** summarizes the estrogen effects on the immune cells that we described in the previous section.

## REGULATION OF IMMUNE CELL FUNCTIONS BY ER INHIBITORS TREATMENT IN BREAST CANCER PATIENTS

It is widely known and accepted that the use of inhibitors of ER in the treatment of patients with estrogen-positive breast cancer has offered high survival rates (161). However, their use in other breast cancer phenotypes or their effects on the immune system cells in clinical stages have not been addressed.

In the previous section, we described several in *in vitro* and *in vivo* data that clearly show how the tumor immune infiltrate cells could play an important role in the development, progression, and response of breast cancer through ERs signaling activation. However, they also encourage to focus on the modulation of their antitumor functions with ER inhibitors. In this regard, few studies have reported the effect of ER inhibitors on immune infiltrating breast tumor cell functions in clinical phases. As we described previously, Tregs have been found to be up-modulated in breast tumors, and high number of these cells were present in high-grade ER-negative breast cancer patients. Also, they were related with ER-positive breast tumors identified with high-risk patients (7, 162). It is known that Tregs give valuable information about breast cancer prognosis and progression, since high number of Tregs can identify patients at risk of relapse after 5 years. Nevertheless, there was not a relationship between Tregs number and the type of therapy that patients received (7). Interestingly, in 2009, Generali et al. reported that Tregs number were significant decreased in patients who received an aromatase inhibitor treatment alone (letrozole) and in combination with an antineoplastic agent (letrozole + cyclophosphamide) (163). Another *in vivo* model reported that ICI 182,780 could reverse the estradiol actions for inducing Tregs phenotype (154). These facts can indicate that E2 inhibition is an important antitumor strategy for manipulating the tumor microenvironment through of inhibiting the function and number of Tregs, besides that letrozole also might be useful in combination treatments in patients with ER-negative tumors regardless of ER expression into the tumor cells. Returning to the fact that this hormone can inhibit the NK activity, an interesting work reported that post-menopausal stage I breast cancer patients who received tamoxifen for 1 month shown a statistically significant increase in NK activity, however, the NK activity could not be related with the ER expression in breast

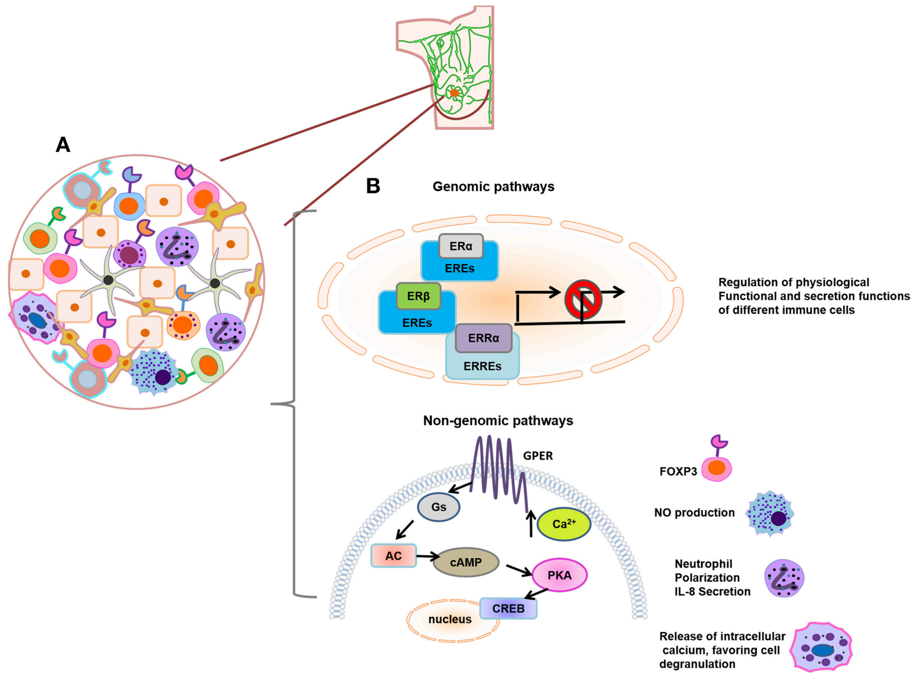


**FIGURE 3 | (A)** Schematic representation of immune infiltrating tumor cells. **(B)** Genomic and non-genomic estrogen pathways on immune cells. Estrogen regulates physiological, functional and secretion actions of different immune cells; these effects are mainly studied by the activation of genomic pathways such as ERα, ERβ, or ERRα. In addition, little effects of Tregs (increase of Foxp3 expression), macrophages (NO production), neutrophils (neutrophil polarization and IL-8 secretion), and mast cells (movilization of intracellular calcium, favoring cell degranulation) have been described by the action of non-genomic pathway.

**TABLE 1 |** Estradiol effects of different immune cells.

| Type of immune cells | Modulation | References |
|---|---|---|
| DCs | Increase expression of co-stimulatory molecules such as INF-γ<br>Stimulation of T-cell proliferation and differentiation<br>Induction of pro-inflammatory cytokines and chemokines; TNFα, IL-2, IL-6, IL-10, IL-8, MCP-1<br>DCs migratory response to lymph nodes after LPS stimulation<br>Induction of DCs differentiation via GM-CSF and the IRF4<br>Generation of tolerogenic DCs affecting their cell antigen presenting function | (39–47) |
| Macrophages | Stimulates the NO release<br>Modulate the lipid metabolism of macrophages through the release of arachidonic acid and prostaglandin E2 production<br>Modulates catalase CAT activity<br>Reduced MMP-9 expression<br>Increase macrophage survival through Bcl-2 activation<br>Reduced the IL-8 expression<br>Decrease IL-6, TNF-α, IL-1β expression<br>Reduce the TNF-α gene expression<br>Induce alternative macrophage activation through of the modulation of activity and expression of several markers such as Fizz1, Ym1 and arginase 1, CD163 and CD206 | (49–81) |
| Mast cells | Induction of histamine, leukotriene, β-hexosaminidase and tryptase release<br>Induction of chemokine receptors (CCR4 and CCR5)<br>Release of intracellular calcium- favoring degranulation | (10, 85–94) |
| Neutrophils | Enhances the NO production and the neuronal nitric oxide synthase<br>Promotes neutrophil pro-inflammatory phenotype through GPER- cAMP/PKA/CREB, MAPK activation<br>Increase IL-1β, IL-8, PTGS2, SOCS3, and G-CSF gene expression<br>Increase the IL-8 release via G1/GPER<br>Upregulate of two markers of neutrophil activation (CD11b and CD62L)<br>Reduce the IL-8 neutrophil release and the CD62L expression via ERα<br>Reduced the neutrophil chemotaxis and the superoxide anion production<br>Increase the number of neutrophils in different lymphoid tissues and the NSPs including NE, PR3, and CG<br>Increase the MPO expression | (100–114) |
| NK cells | Reduction of NK cells cytotoxic activity in long time of exposure<br>Enhancement of tumor susceptibility and metastasis<br>Stimulation of NK cells activity in short time of exposure<br>Induction of PI-9 | (118–126) |
| B lymphocytes | Enhancement the IgG and IgM production<br>Increase the survival, proliferation, migration and chemotaxis | (39, 128–136) |
| $TCD4^+$ and $TCD8^+$Th1Th2 | Promotion of $CD4^+/CD8^+$ T phenotype expression<br>Induction of glycolytic genes implicated in the inflammatory cytokine production and T cell proliferation via ERRα<br>Inhibition of pro-inflammatory cytokines IL-2, IL-12, IFN-γ and TNF-α<br>Negative regulation of IFNγ promoter<br>Increment of IL-10, IL-4, and TGF-β<br>Induction of Th2 transcription factor GATA-3 | (37, 38, 113, 138–150) |
| Tregs | Induction of FoxP3 and IL-10 gene expression<br>Maintenance of Tregs phenotype<br>Activation of FoxP3 activity via estradiol-ERα-EREs<br>Induction of FoxP3, PD-1 and CTLA-4 protein expression via GPER<br>Increase of immuno-modulatory cytokines such as TGF-β and IL-10 | (153–160) |

tumors due to the limiting number of patients included (164), this fact correlates with mice models and the estrogen actions in NK cells activation (124). Besides, it is important to mention that some studies have reported a low proportion of NK cells into late stages of breast tumors (165); therefore, the work of Berry et al. suggests that in the early stages of breast cancer, the patients treated with tamoxifen could be benefited for activate the NK cells instead of the use of this drug in late stages, concluding that these cells could be consider as therapeutic targets.

With respect to E2 modulation on the TAMs function, there are not reports that have evaluated its inhibition effect in clinical trials. We described before that E2 promotes the alternative macrophage activation (72, 74). Interestingly, Hollmén et al. found that ER positive and ER negative tumors induced different macrophage phenotypes with different biological functions, morphology and cytokine and chemokine secretion. In fact, alternatively activated macrophages present in triple negative breast cancer have a down-regulation in citrulline metabolism (80). From this concept, it would be interesting to study the effects of this hormone on the citrulline metabolism, since it is known that the nitric oxide synthase (iNOS) expression is enhanced by E2 action (166) and simultaneously, this enzyme

is related with citrulline and arginine metabolism, determining the macrophages activation phenotype in breast cancer (167). Neutrophils number *in situ* into breast tumors are associated with poor prognosis (102), so that, he modulation of their number could interesting for breast cancer patients. In 2017, Dai et al. clearly demonstrated that estradiol treatment increased the neutrophils number in spleens of mice (105). An increased neutrophil number was also found in complete blood of prostate cancer patients treated with estramustine (168), an antineoplastic agent with ERs affinity (169). However, at present there are not reports about the neutrophil modulation into breast cancer tumors by ER inhibitors. On the other hand, it is known that in neutrophils, NETs formation is relevant for pathogen death, a selective estrogen receptor modulator (SERM), raloxifene, inhibited NETs formation of human neutrophils, interfering with bacteria clearance, after the treatment of the NET inducer phorbol 12-myristate 13-acetate (PMA) (170); opposite effect to that found with tamoxifen treatment (171). With respect to other immune populations, there are not reports about their function modulation by ER inhibitors in breast cancer patients. In addition to the data described above, our workgroup reported that endocrine disrupting compounds such as bisphenol A (BPA) have a significant effect in the modulation of ERα expression in T lymphocytes, macrophages and NK cells of breast cancer tumors as well as in the tumor growth. Impressively, a single administration of BPA in neo-natal mice resulted in an important changes in the Tregs presence infiltrated into breast tumors in the adult stage (172). These facts provide new approaches to study the effect of various compounds with estrogenic activity on the modulation of immune cells as well as in the selective inhibition of ER.

On the other hand, although different immunohistochemical studies as well as DNA sequencing data have given promising landscapes of infiltrating immune cells in this neoplasm for its therapy (14, 21), and despite the extraordinary efforts to reach a consensus on the study of the invasive population in breast cancer in the daily histopathological practice (173, 174), different techniques such as flow cytometry must be applied in the clinic in order to guarantee precise studies; since it has been described that according to the tumor area the presence of infiltrating lymphocytes can vary (175). The above would allow offering personalized, predictive, and effective combined breast cancer treatments.

## CONCLUDING REMARKS AND FUTURE DIRECTIONS

The main aim of this paper is to stand out the components of immune cells within the tumor microenvironment in different phenotypes of breast cancer, and the participation of the E2 and its receptors in their function. As described above, E2 modifies the functions of different immune populations, although the effects of this hormone were described in a particular way in each immune lineage, it is known that all of them are interconnected by cytokines, maintaining a dynamic interaction into the tumor microenvironment. Several reports have mentioned that immune infiltrating cells are good players for avoiding the progression of breast cancer and have a significant clinical impact in the response to the treatment in an independent manner of the cancer phenotype (176, 177). However, little is known about their percentage and their grade of activation or anergy in different advanced clinic stages of this pathology, which might be modified due to the intratumoral E2 concentration. Based on the role that E2 and its signaling have in different populations of the immune system, we consider important to evaluate or measure the intratumoral levels of this hormone and/or different compounds such as endocrine disruptors mainly in advanced stages of this disease, which could be associated with their pro-anergic state. It has been documented that the concentration of E2 as well as the enzyme that produces it (aromatase) are elevated inside the tumor (178, 179), affecting not only epithelial cells growth but also the immune cell effects. Taking into consideration the previous fact, we also regard the use of intratumoral therapy using ER inhibitors in the different types of breast cancer as an integral adjuvant approach for heightening both other therapies and the immune response. The previous concept has taken on importance in cancer therapy, indeed, new studies on this topic are being done with different treatment schemes (180).Finally, the immune cells function and their cytokines are key factors whose modulation strongly encouraged to study and consider them as predictive markers and important therapy targets in different subtypes of breast cancer.

## AUTHOR CONTRIBUTIONS

MS-M was in charge of all compilation of information, drafting of the manuscript, and participated in its conception. JM-M participated in the critical revision of the content of the manuscript and made a substantive intellectual contribution to drafting it. All authors read and approved the final manuscript.

## FUNDING

Financial support: Grant IN-209719 from Programa de Apoyo a Proyectos de Investigación e Innovación Tecnológica (PAPIIT), Dirección General de Asuntos del Personal Académico (DGAPA), Universidad Nacional Autónoma de México (UNAM) and Grant 2125 Programa Fronteras en la Ciencia del Consejo Nacional de Ciencia y Tecnología (CONACYT) both to JM-M. MS-M receives a Post-Doctoral fellowship from DGAPA, UNAM.

## REFERENCES

1. Ahn SG, Kim SJ, Kim C, Jeong J. Molecular classification of triple-negative breast cancer. *J Breast Cancer.* (2016) 19:223–30. doi: 10.4048/jbc.2016.19.3.223
2. Onland-Moret NC, Kaaks R, van Noord PA, Rinaldi S, Key T, Grobbee DE, et al. Urinary endogenous sex hormone levels and the risk of postmenopausal breast cancer. *Br J Cancer.* (2003) 88:1394–9. doi: 10.1038/sj.bjc.6600890
3. Coughlin SS, Ekwueme DU. Breast cancer as a global health concern. *Cancer Epidemiol.* (2009) 33:315–8. doi: 10.1016/j.canep.2009.10.003

4. Schnitt SJ. Classification and prognosis of invasive breast cancer: from morphology to molecular taxonomy. *Mod Pathol.* (2010) 23(Suppl. 2):S60–64. doi: 10.1038/modpathol.2010.33
5. Polyak K. Heterogeneity in breast cancer. *J Clin Invest.* (2011) 121:3786–8. doi: 10.1172/JCI60534
6. Esquivel-Velazquez M, Ostoa-Saloma P, Palacios-Arreola MI, Nava-Castro KE, Castro JI, Morales-Montor J. The role of cytokines in breast cancer development and progression. *J Interferon Cytokine Res.* (2015) 35:1–16. doi: 10.1089/jir.2014.0026
7. Bates GJ, Fox SB, Han C, Leek RD, Garcia JF, Harris AL, et al. Quantification of regulatory T cells enables the identification of high-risk breast cancer patients and those at risk of late relapse. *J Clin Oncol.* (2006) 24:5373–80. doi: 10.1200/JCO.2006.05.9584
8. Wang M, Zhang C, Song Y, Wang Z, Wang Y, Luo F, et al. Mechanism of immune evasion in breast cancer. *Onco Targets Ther.* (2017) 10:1561–73. doi: 10.2147/OTT.S126424
9. Glajcar A, Szpor J, Pacek A, Tyrak KE, Chan F, Streb J, et al. The relationship between breast cancer molecular subtypes and mast cell populations in tumor microenvironment. *Virchows Arch.* (2017) 470:505–15. doi: 10.1007/s00428-017-2103-5
10. Aponte-Lopez A, Fuentes-Panana EM, Cortes-Munoz D, Munoz-Cruz S. Mast cell, the neglected member of the tumor microenvironment: role in breast cancer. *J Immunol Res.* (2018) 2018:2584243. doi: 10.1155/2018/2584243
11. Sica A, Larghi P, Mancino A, Rubino L, Porta C, Totaro MG, et al. Macrophage polarization in tumour progression. *Semin Cancer Biol.* (2008) 18:349–55. doi: 10.1016/j.semcancer.2008.03.004
12. de Kruijf EM, Sajet A, van Nes JG, Putter H, Smit VT, Eagle RA, et al. NKG2D ligand tumor expression and association with clinical outcome in early breast cancer patients: an observational study. *BMC Cancer.* (2012) 12:24. doi: 10.1186/1471-2407-12-24
13. Ali HR, Chlon L, Pharoah PD, Markowetz F, Caldas C. Patterns of immune infiltration in breast cancer and their clinical implications: a gene-expression-based retrospective study. *PLoS Med.* (2016) 13:e1002194. doi: 10.1371/journal.pmed.1002194
14. Hammerl D, Smid M, Timmermans AM, Sleijfer S, Martens JWM, Debets R. Breast cancer genomics and immuno-oncological markers to guide immune therapies. *Semin Cancer Biol.* (2018) 52(Pt 2):178–88. doi: 10.1016/j.semcancer.2017.11.003
15. Denkert C, Loibl S, Noske A, Roller M, Muller BM, Komor M, et al. Tumor-associated lymphocytes as an independent predictor of response to neoadjuvant chemotherapy in breast cancer. *J Clin Oncol.* (2010) 28:105–13. doi: 10.1200/JCO.2009.23.7370
16. Watanabe MA, Oda JM, Amarante MK, Cesar Voltarelli J. Regulatory T cells and breast cancer: implications for immunopathogenesis. *Cancer Metastasis Rev.* (2010) 29:569–79. doi: 10.1007/s10555-010-9247-y
17. Mahmoud SM, Paish EC, Powe DG, Macmillan RD, Grainge MJ, Lee AH, et al. Tumor-infiltrating CD8+ lymphocytes predict clinical outcome in breast cancer. *J Clin Oncol.* (2011) 29:1949–55. doi: 10.1200/JCO.2010.30.5037
18. Medrek C, Ponten F, Jirstrom K, Leandersson K. The presence of tumor associated macrophages in tumor stroma as a prognostic marker for breast cancer patients. *BMC Cancer.* (2012) 12:306. doi: 10.1186/1471-2407-12-306
19. Ali HR, Provenzano E, Dawson SJ, Blows FM, Liu B, Shah M, et al. Association between CD8+ T-cell infiltration and breast cancer survival in 12,439 patients. *Ann Oncol.* (2014) 25:1536–43. doi: 10.1093/annonc/mdu191
20. Svensson S, Abrahamsson A, Rodriguez GV, Olsson AK, Jensen L, Cao Y, et al. CCL2 and CCL5 are novel therapeutic targets for estrogen-dependent breast cancer. *Clin Cancer Res.* (2015) 21:3794–805. doi: 10.1158/1078-0432.CCR-15-0204
21. Dieci MV, Tsvetkova V, Orvieto E, Piacentini F, Ficarra G, Griguolo G, et al. Immune characterization of breast cancer metastases: prognostic implications. *Breast Cancer Res.* (2018) 20:62. doi: 10.1186/s13058-018-1003-1
22. Ma C, Zhang Q, Ye J, Wang F, Zhang Y, Wevers E, et al. Tumor-infiltrating gammadelta T lymphocytes predict clinical outcome in human breast cancer. *J Immunol.* (2012) 189:5029–36. doi: 10.4049/jimmunol.1201892
23. Ethier JL, Desautels D, Templeton A, Shah PS, Amir E. Prognostic role of neutrophil-to-lymphocyte ratio in breast cancer: a systematic review and meta-analysis. *Breast Cancer Res.* (2017) 19:2. doi: 10.1186/s13058-016-0794-1
24. Ascenzi P, Bocedi A, Marino M. Structure-function relationship of estrogen receptor alpha and beta: impact on human health. *Mol Aspects Med.* (2006) 27:299–402. doi: 10.1016/j.mam.2006.07.001
25. Albanito L, Sisci D, Aquila S, Brunelli E, Vivacqua A, Madeo A, et al. Epidermal growth factor induces G protein-coupled receptor 30 expression in estrogen receptor-negative breast cancer cells. *Endocrinology.* (2008) 149:3799–808. doi: 10.1210/en.2008-0117
26. Ruan SQ, Wang SW, Wang ZH, Zhang SZ. Regulation of HRG-beta1-induced proliferation, migration and invasion of MCF-7 cells by upregulation of GPR30 expression. *Mol Med Rep.* (2012) 6:131–8. . doi: 10.3892/mmr.2012.874
27. Prossnitz ER, Arterburn JB. International union of basic and clinical pharmacology. XCVII G protein-coupled estrogen receptor and its pharmacologic modulators. *Pharmacol Rev.* (2015) 67:505–40. doi: 10.1124/pr.114.009712
28. Evans NJ, Bayliss AL, Reale V, Evans PD. Characterisation of signalling by the endogenous GPER1 (GPR30) Receptor in an embryonic mouse hippocampal cell line (mHippoE-18). *PLoS ONE.* (2016) 11:e0152138. doi: 10.1371/journal.pone.0152138
29. Frasor J, Danes JM, Komm B, Chang KC, Lyttle CR, Katzenellenbogen BS. Profiling of estrogen up- and down-regulated gene expression in human breast cancer cells: insights into gene networks and pathways underlying estrogenic control of proliferation and cell phenotype. *Endocrinology.* (2003) 144:4562–74. doi: 10.1210/en.2003-0567
30. Strom A, Hartman J, Foster JS, Kietz S, Wimalasena J, Gustafsson JA. Estrogen receptor beta inhibits 17beta-estradiol-stimulated proliferation of the breast cancer cell line T47D. *Proc Natl Acad Sci USA.* (2004) 101:1566–71. doi: 10.1073/pnas.0308319100
31. Nutter LM, Wu YY, Ngo EO, Sierra EE, Gutierrez PL, Abul-Hajj YJ. An o-quinone form of estrogen produces free radicals in human breast cancer cells: correlation with DNA damage. *Chem Res Toxicol.* (1994) 7:23–8. doi: 10.1021/tx00037a004
32. Mobley JA, Brueggemeier RW. Estrogen receptor-mediated regulation of oxidative stress and DNA damage in breast cancer. *Carcinogenesis.* (2004) 25:3–9. doi: 10.1093/carcin/bgg175
33. Cavalieri EL, Rogan EG. Unbalanced metabolism of endogenous estrogens in the etiology and prevention of human cancer. *J Steroid Biochem Mol Biol.* (2011) 125:169–80. doi: 10.1016/j.jsbmb.2011.03.008
34. Elkin M, Orgel A, Kleinman HK. An angiogenic switch in breast cancer involves estrogen and soluble vascular endothelial growth factor receptor 1. *J Natl Cancer Inst.* (2004) 96:875–8. doi: 10.1093/jnci/djh140
35. Chaudhary S, Madhukrishna B, Adhya AK, Keshari S, Mishra SK. Overexpression of caspase 7 is ERalpha dependent to affect proliferation and cell growth in breast cancer cells by targeting p21(Cip). *Oncogenesis.* (2016) 5:e219. doi: 10.1038/oncsis.2016.12
36. Lannigan DA. Estrogen receptor phosphorylation. *Steroids.* (2003) 68:1–9. doi: 10.1016/S0039-128X(02)00110-1
37. Cunningham M, Gilkeson G. Estrogen receptors in immunity and autoimmunity. *Clin Rev Allergy Immunol.* (2011) 40:66–73. doi: 10.1007/s12016-010-8203-5
38. Khan D, Ansar Ahmed S. The immune system is a natural target for estrogen action: opposing effects of estrogen in two prototypical autoimmune diseases. *Front Immunol.* (2015) 6:635. doi: 10.3389/fimmu.2015.00635
39. Kovats S. Estrogen receptors regulate innate immune cells and signaling pathways. *Cell Immunol.* (2015) 294:63–9. doi: 10.1016/j.cellimm.2015.01.018
40. Bengtsson AK, Ryan EJ, Giordano D, Magaletti DM, Clark EA. 17beta-estradiol (E2) modulates cytokine and chemokine expression in human monocyte-derived dendritic cells. *Blood.* (2004) 104:1404–10. doi: 10.1182/blood-2003-10-3380
41. Liu HY, Buenafe AC, Matejuk A, Ito A, Zamora A, Dwyer J, et al. Estrogen inhibition of EAE involves effects on dendritic cell function. *J Neurosci Res.* (2002) 70:238–48. doi: 10.1002/jnr.10409

42. Paharkova-Vatchkova V, Maldonado R, Kovats S. Estrogen preferentially promotes the differentiation of CD11c+ CD11b(intermediate) dendritic cells from bone marrow precursors. *J Immunol.* (2004) 172:1426–36. doi: 10.4049/jimmunol.172.3.1426
43. Mao A, Paharkova-Vatchkova V, Hardy J, Miller MM, Kovats S. Estrogen selectively promotes the differentiation of dendritic cells with characteristics of Langerhans cells. *J Immunol.* (2005) 175:5146–51. doi: 10.4049/jimmunol.175.8.5146
44. Nalbandian G, Paharkova-Vatchkova V, Mao A, Nale S, Kovats S. The selective estrogen receptor modulators, tamoxifen and raloxifene, impair dendritic cell differentiation and activation. *J Immunol.* (2005) 175:2666–75. doi: 10.4049/jimmunol.175.4.2666
45. Carreras E, Turner S, Frank MB, Knowlton N, Osban J, Centola M, et al. Estrogen receptor signaling promotes dendritic cell differentiation by increasing expression of the transcription factor IRF4. *Blood.* (2010) 115:238–46. doi: 10.1182/blood-2009-08-236935
46. Zhang J, Ye ZW, Chen W, Manevich Y, Mehrotra S, Ball LE, et al. S-Glutathionylation of estrogen receptor alpha affects dendritic cell function. *J Biol Chem*. (2018) 293:4366–80. doi: 10.1074/jbc.M117.814327
47. Papenfuss TL, Powell ND, McClain MA, Bedarf A, Singh A, Gienapp IE, et al. Estriol generates tolerogenic dendritic cells *in vivo* that protect against autoimmunity. *J Immunol.* (2011) 186:3346–55. doi: 10.4049/jimmunol.1001322
48. Mantovani A, Sozzani S, Locati M, Allavena P, Sica A. Macrophage polarization: tumor-associated macrophages as a paradigm for polarized M2 mononuclear phagocytes. *Trends Immunol.* (2002) 23:549–55. doi: 10.1016/S1471-4906(02)02302-5
49. Weusten JJ, Blankenstein MA, Gmelig-Meyling FH, Schuurman HJ, Kater L, Thijssen JH. Presence of oestrogen receptors in human blood mononuclear cells and thymocytes. *Acta Endocrinol.* (1986) 112:409–14. doi: 10.1530/acta.0.1120409
50. Murphy AJ, Guyre PM, Wira CR, Pioli PA. Estradiol regulates expression of estrogen receptor ERalpha46 in human macrophages. *PLoS ONE.* (2009) 4:e5539. doi: 10.1371/journal.pone.0005539
51. Mor G, Sapi E, Abrahams VM, Rutherford T, Song J, Hao XY, et al. Interaction of the estrogen receptors with the Fas ligand promoter in human monocytes. *J Immunol.* (2003) 170:114–22. doi: 10.4049/jimmunol.170.1.114
52. Harkonen PL, Vaananen HK. Monocyte-macrophage system as a target for estrogen and selective estrogen receptor modulators. *Ann N Y Acad Sci.* (2006) 1089:218–27. doi: 10.1196/annals.1386.045
53. MacMicking J, Xie QW, Nathan C. Nitric oxide and macrophage function. *Annu Rev Immunol.* (1997) 15:323–50. doi: 10.1146/annurev.immunol.15.1.323
54. Stefano GB, Prevot V, Beauvillain JC, Fimiani C, Welters I, Cadet P, et al. Estradiol coupling to human monocyte nitric oxide release is dependent on intracellular calcium transients: evidence for an estrogen surface receptor. *J Immunol.* (1999) 163:3758–63.
55. Azenabor AA, Yang S, Job G, Adedokun OO. Expression of iNOS gene in macrophages stimulated with 17beta-estradiol is regulated by free intracellular $Ca2+$. *Biochem Cell Biol.* (2004) 82:381–90. doi: 10.1139/o04-032
56. Curuvija I, Stanojevic S, Arsenovic-Ranin N, Blagojevic V, Dimitrijevic M, Vidic-Dankovic B, et al. Sex differences in macrophage functions in middle-aged rats: relevance of estradiol level and macrophage estrogen receptor expression. *Inflammation.* (2017) 40:1087–101. doi: 10.1007/s10753-017-0551-3
57. Netea-Maier RT, Smit JWA, Netea MG. Metabolic changes in tumor cells and tumor-associated macrophages: a mutual relationship. *Cancer Lett.* (2018) 413:102–9. doi: 10.1016/j.canlet.2017.10.037
58. Lu B, Jiang YJ, Choy PC. 17-Beta estradiol enhances prostaglandin E2 production in human U937-derived macrophages. *Mol Cell Biochem.* (2004) 262:101–10. doi: 10.1023/B:MCBI.0000038222.08915.84
59. Tan HY, Wang N, Li S, Hong M, Wang X, Feng Y. The reactive oxygen species in macrophage polarization: reflecting its dual role in progression and treatment of human diseases. *Oxid Med Cell Longev.* (2016) 2016:2795090. doi: 10.1155/2016/2795090
60. Azevedo RB, Lacava ZG, Miyasaka CK, Chaves SB, Curi R. Regulation of antioxidant enzyme activities in male and female rat macrophages by sex steroids. *Braz J Med Biol Res.* (2001) 34:683–7. doi: 10.1590/S0100-879X2001000500018
61. Vegeto E, Ghisletti S, Meda C, Etteri S, Belcredito S, Maggi A. Regulation of the lipopolysaccharide signal transduction pathway by 17beta-estradiol in macrophage cells. *J Steroid Biochem Mol Biol.* (2004) 91:59–66. doi: 10.1016/j.jsbmb.2004.02.004
62. Subramanian M, Shaha C. Up-regulation of Bcl-2 through ERK phosphorylation is associated with human macrophage survival in an estrogen microenvironment. *J Immunol.* (2007) 179:2330–8. doi: 10.4049/jimmunol.179.4.2330
63. Subramanian M, Shaha C. Oestrogen modulates human macrophage apoptosis via differential signalling through oestrogen receptor-alpha and beta. *J Cell Mol Med.* (2009) 13:2317–29. doi: 10.1111/j.1582-4934.2008.00679.x
64. Takeda K, Akira S. Toll-like receptors in innate immunity. *Int Immunol.* (2005) 17:1–14. doi: 10.1093/intimm/dxh186
65. Pioli PA, Jensen AL, Weaver LK, Amiel E, Shen Z, Shen L, et al. Estradiol attenuates lipopolysaccharide-induced CXC chemokine ligand 8 production by human peripheral blood monocytes. *J Immunol.* (2007) 179:6284–90. doi: 10.4049/jimmunol.179.9.6284
66. Turner MD, Nedjai B, Hurst T, Pennington DJ. Cytokines and chemokines: at the crossroads of cell signalling and inflammatory disease. *Biochim Biophys Acta.* (2014) 1843:2563–82. doi: 10.1016/j.bbamcr.2014.05.014
67. Ralston SH, Russell RG, Gowen M. Estrogen inhibits release of tumor necrosis factor from peripheral blood mononuclear cells in postmenopausal women. *J Bone Miner Res.* (1990) 5:983–8. doi: 10.1002/jbmr.5650050912
68. Lin SC, Yamate T, Taguchi Y, Borba VZ, Girasole G, O'Brien CA, et al. Regulation of the gp80 and gp130 subunits of the IL-6 receptor by sex steroids in the murine bone marrow. *J Clin Invest.* (1997) 100:1980–90. doi: 10.1172/JCI119729
69. Rogers A, Eastell R. The effect of 17beta-estradiol on production of cytokines in cultures of peripheral blood. *Bone.* (2001) 29:30–4. doi: 10.1016/S8756-3282(01)00468-9
70. Kramer PR, Kramer SF, Guan G. 17 beta-estradiol regulates cytokine release through modulation of CD16 expression in monocytes and monocyte-derived macrophages. *Arthritis Rheum.* (2004) 50:1967–75. doi: 10.1002/art.20309
71. Srivastava S, Weitzmann MN, Cenci S, Ross FP, Adler S, Pacifici R. Estrogen decreases TNF gene expression by blocking JNK activity and the resulting production of c-Jun and JunD. *J Clin Invest.* (1999) 104:503–13. doi: 10.1172/JCI7094
72. Routley CE, Ashcroft GS. Effect of estrogen and progesterone on macrophage activation during wound healing. *Wound Repair Regen.* (2009) 17:42–50. doi: 10.1111/j.1524-475X.2008.00440.x
73. Mosser DM, Edwards JP. Exploring the full spectrum of macrophage activation. *Nat Rev Immunol.* (2008) 8:958–69. doi: 10.1038/nri2448
74. Campbell L, Emmerson E, Williams H, Saville CR, Krust A, Chambon P, et al. Estrogen receptor-alpha promotes alternative macrophage activation during cutaneous repair. *J Invest Dermatol.* (2014) 134:2447–57. doi: 10.1038/jid.2014.175
75. Toniolo A, Fadini GP, Tedesco S, Cappellari R, Vegeto E, Maggi A, et al. Alternative activation of human macrophages is rescued by estrogen treatment in vitro and impaired by menopausal status. *J Clin Endocrinol Metab.* (2015) 100:E50–58. doi: 10.1210/jc.2014-2751
76. Leek RD, Lewis CE, Whitehouse R, Greenall M, Clarke J, Harris AL. Association of macrophage infiltration with angiogenesis and prognosis in invasive breast carcinoma. *Cancer Res.* (1996) 56:4625–9.
77. Hanada T, Nakagawa M, Emoto A, Nomura T, Nasu N, Nomura Y. Prognostic value of tumor-associated macrophage count in human bladder cancer. *Int J Urol.* (2000) 7:263–9. doi: 10.1046/j.1442-2042.2000.00190.x
78. Lissbrant IF, Stattin P, Wikstrom P, Damber JE, Egevad L, Bergh A. Tumor associated macrophages in human prostate cancer: relation to clinicopathological variables and survival. *Int J Oncol.* (2000) 17:445–51. doi: 10.3892/ijo.17.3.445
79. Erreni M, Mantovani A, Allavena P. Tumor-associated Macrophages (TAM) and inflammation in colorectal cancer. *Cancer Microenviron.* (2011) 4:141–54. doi: 10.1007/s12307-010-0052-5

80. Hollmen M, Roudnicky F, Karaman S, Detmar M. Characterization of macrophage–cancer cell crosstalk in estrogen receptor positive and triple-negative breast cancer. *Sci Rep.* (2015) 5:9188. doi: 10.1038/srep09188
81. Ning C, Xie B, Zhang L, Li C, Shan W, Yang B, et al. Infiltrating macrophages induce eralpha expression through an IL17A-mediated epigenetic mechanism to sensitize endometrial cancer cells to estrogen. *Cancer Res.* (2016) 76:1354–66. doi: 10.1158/0008-5472.CAN-15-1260
82. Krystel-Whittemore M, Dileepan KN, Wood JG. Mast cell: a multi-functional master cell. *Front Immunol.* (2015) 6:620. doi: 10.3389/fimmu.2015.00620
83. Irani AA, Schechter NM, Craig SS, DeBlois G, Schwartz LB. Two types of human mast cells that have distinct neutral protease compositions. *Proc Natl Acad Sci USA.* (1986) 83:4464–8. doi: 10.1073/pnas.83.12.4464
84. Cimpean AM, Tamma R, Ruggieri S, Nico B, Toma A, Ribatti D. Mast cells in breast cancer angiogenesis. *Crit Rev Oncol Hematol.* (2017) 115:23–6. doi: 10.1016/j.critrevonc.2017.04.009
85. Zhao XJ, McKerr G, Dong Z, Higgins CA, Carson J, Yang ZQ, et al. Expression of oestrogen and progesterone receptors by mast cells alone, but not lymphocytes, macrophages or other immune cells in human upper airways. *Thorax.* (2001) 56:205–11. doi: 10.1136/thorax.56.3.205
86. Jiang YA, Zhang YY, Luo HS, Xing SF. Mast cell density and the context of clinicopathological parameters and expression of p185, estrogen receptor, and proliferating cell nuclear antigen in gastric carcinoma. *World J Gastroenterol.* (2002) 8:1005–8. doi: 10.3748/wjg.v8.i6.1005
87. Nicovani S, Rudolph MI. Estrogen receptors in mast cells from arterial walls. *Biocell.* (2002) 26:15–24.
88. Zierau O, Zenclussen AC, Jensen F. Role of female sex hormones, estradiol and progesterone, in mast cell behavior. *Front Immunol.* (2012) 3:169. doi: 10.3389/fimmu.2012.00169
89. O'Brien E, Dolinoy DC, Mancuso P. Bisphenol A at concentrations relevant to human exposure enhances histamine and cysteinyl leukotriene release from bone marrow-derived mast cells. *J Immunotoxicol.* (2014) 11:84–9. doi: 10.3109/1547691X.2013.800925
90. Medina V, Croci M, Crescenti E, Mohamad N, Sanchez-Jimenez F, Massari N, et al. The role of histamine in human mammary carcinogenesis: H3 and H4 receptors as potential therapeutic targets for breast cancer treatment. *Cancer Biol Ther.* (2008) 7:28–35. doi: 10.4161/cbt.7.1.5123
91. Cocchiara R, Albeggiani G, Di Trapani G, Azzolina A, Lampiasi N, Rizzo F, et al. Oestradiol enhances in vitro the histamine release induced by embryonic histamine-releasing factor (EHRF) from uterine mast cells. *Hum Reprod.* (1992) 7:1036–41. doi: 10.1093/oxfordjournals.humrep.a137790
92. Narita S, Goldblum RM, Watson CS, Brooks EG, Estes DM, Curran EM, et al. Environmental estrogens induce mast cell degranulation and enhance IgE-mediated release of allergic mediators. *Environ Health Perspect.* (2007) 115:48–52. doi: 10.1289/ehp.9378
93. Mangia A, Malfettone A, Rossi R, Paradiso A, Ranieri G, Simone G, et al. Tissue remodelling in breast cancer: human mast cell tryptase as an initiator of myofibroblast differentiation. *Histopathology.* (2011) 58:1096–106. doi: 10.1111/j.1365-2559.2011.03842.x
94. Jensen F, Woudwyk M, Teles A, Woidacki K, Taran F, Costa S, et al. Estradiol and progesterone regulate the migration of mast cells from the periphery to the uterus and induce their maturation and degranulation. *PLoS ONE.* (2010) 5:e14409. doi: 10.1371/journal.pone.0014409
95. Zaitsu M, Narita S, Lambert KC, Grady JJ, Estes DM, Curran EM, et al. Estradiol activates mast cells via a non-genomic estrogen receptor-alpha and calcium influx. *Mol Immunol.* (2007) 44:1977–85. doi: 10.1016/j.molimm.2006.09.030
96. Mayadas TN, Cullere X, Lowell CA. The multifaceted functions of neutrophils. *Annu Rev Pathol.* (2014) 9:181–218. doi: 10.1146/annurev-pathol-020712-164023
97. Rosales C, Demaurex N, Lowell CA, Uribe-Querol E. Neutrophils: their role in innate and adaptive immunity. *J Immunol Res.* (2016) 2016:1469780. doi: 10.1155/2016/1469780
98. Tecchio C, Micheletti A, Cassatella MA. Neutrophil-derived cytokines: facts beyond expression. *Front Immunol.* (2014) 5:508. doi: 10.3389/fimmu.2014.00508
99. Stygar D, Westlund P, Eriksson H, Sahlin L. Identification of wild type and variants of oestrogen receptors in polymorphonuclear and mononuclear leucocytes. *Clin Endocrinol.* (2006) 64:74–81. doi: 10.1111/j.1365-2265.2005.02420.x
100. Molloy EJ, O'Neill AJ, Grantham JJ, Sheridan-Pereira M, Fitzpatrick JM, Webb DW, et al. Sex-specific alterations in neutrophil apoptosis: the role of estradiol and progesterone. *Blood.* (2003) 102:2653–9. doi: 10.1182/blood-2003-02-0649
101. Bain BJ, England JM. Normal haematological values: sex difference in neutrophil count. *Br Med J.* (1975) 1:306–9. doi: 10.1136/bmj.1.5953.306
102. Duran MG, Galvez GG, de Frutos T, Diaz Recasens J, Casado S, Lopez Farre A. 17 Beta-estradiol-stimulated nitric oxide production by neutrophils: effect on platelet activation. *Obstet Gynecol.* (2000) 95:284–90. doi: 10.1097/00006250-200002000-00022
103. Molero L, Garcia-Duran M, Diaz-Recasens J, Rico L, Casado S, Lopez-Farre A. Expression of estrogen receptor subtypes and neuronal nitric oxide synthase in neutrophils from women and men: regulation by estrogen. *Cardiovasc Res.* (2002) 56:43–51. doi: 10.1016/S0008-6363(02)00505-9
104. Pham CT. Neutrophil serine proteases fine-tune the inflammatory response. *Int J Biochem Cell Biol.* (2008) 40:1317–33. doi: 10.1016/j.biocel.2007.11.008
105. Dai R, Cowan C, Heid B, Khan D, Liang Z, Pham CT, et al. Neutrophils and neutrophil serine proteases are increased in the spleens of estrogen-treated C57BL/6 mice and several strains of spontaneous lupus-prone mice. *PLoS ONE.* (2017) 12:e0172105. doi: 10.1371/journal.pone.0172105
106. Fridlender ZG, Sun J, Kim S, Kapoor V, Cheng G, Ling L, et al. Polarization of tumor-associated neutrophil phenotype by TGF-beta: "N1" versus "N2" TAN. *Cancer Cell.* (2009) 16:183–94. doi: 10.1016/j.ccr.2009.06.017
107. Rodenas MC, Tamassia N, Cabas I, Calzetti F, Meseguer J, Cassatella MA, et al. G protein-coupled estrogen receptor 1 regulates human neutrophil functions. *Biomedicine Hub.* (2017) 2:2–2. doi: 10.1159/0004 54981
108. Malisorn N, Morales NP, Sanvarinda Y, Wanikiat P. Characterization of estrogen receptor mediating neutrophil function. *Thai J Pharmacol.* (2010) 32:276–80.
109. Simon SI, Burns AR, Taylor AD, Gopalan PK, Lynam EB, Sklar LA, et al. L-selectin (CD62L) cross-linking signals neutrophil adhesive functions via the Mac-1 (CD11b/CD18) beta 2-integrin. *J Immunol.* (1995) 155:1502–14.
110. Bekesi G, Kakucs R, Varbiro S, Racz K, Sprintz D, Feher J, et al. In vitro effects of different steroid hormones on superoxide anion production of human neutrophil granulocytes. *Steroids.* (2000) 65:889–94. doi: 10.1016/S0039-128X(00)00183-5
111. Bekesi G, Tulassay Z, Racz K, Feher J, Szekacs B, Kakucs R, et al. The effect of estrogens on superoxide anion generation by human neutrophil granulocytes: possible consequences of the antioxidant defense. *Gynecol Endocrinol.* (2007) 23:451–4. doi: 10.1080/09513590701483951
112. El-Benna J, Hurtado-Nedelec M, Marzaioli V, Marie JC, Gougerot-Pocidalo MA, Dang PM. Priming of the neutrophil respiratory burst: role in host defense and inflammation. *Immunol Rev.* (2016) 273:180–93. doi: 10.1111/imr.12447
113. Chung HH, Or YZ, Shrestha S, Loh JT, Lim CL, Ong Z, et al. Estrogen reprograms the activity of neutrophils to foster protumoral microenvironment during mammary involution. *Sci Rep.* (2017) 7:46485. doi: 10.1038/srep46485
114. Vazquez Rodriguez G, Abrahamsson A, Jensen LD, Dabrosin C. Estradiol promotes breast cancer cell migration via recruitment and activation of neutrophils. *Cancer Immunol Res.* (2017) 5:234–47. doi: 10.1158/2326-6066.CIR-16-0150
115. Vivier E, Tomasello E, Baratin M, Walzer T, Ugolini S. Functions of natural killer cells. *Nat Immunol.* (2008) 9:503–10. doi: 10.1038/ni1582
116. Garrido F, Cabrera T, Aptsiauri N. "Hard" and "soft" lesions underlying the HLA class I alterations in cancer cells: implications for immunotherapy. *Int J Cancer.* (2010) 127:249–56. doi: 10.1002/ijc.25270
117. Roberti MP, Mordoh J, Levy EM. Biological role of NK cells and immunotherapeutic approaches in breast cancer. *Front Immunol.* (2012) 3:375. doi: 10.3389/fimmu.2012.00375
118. Seaman WE, Gindhart TD. Effect of estrogen on natural killer cells. *Arthritis Rheum.* (1979) 22:1234–40. doi: 10.1002/art.1780221110
119. Nilsson N, Carlsten H. Estrogen induces suppression of natural killer cell cytotoxicity and augmentation of polyclonal B cell activation. *Cell Immunol.* (1994) 158:131–9. doi: 10.1006/cimm.1994.1262

120. Albrecht AE, Hartmann BW, Scholten C, Huber JC, Kalinowska W, Zielinski CC. Effect of estrogen replacement therapy on natural killer cell activity in postmenopausal women. *Maturitas.* (1996) 25:217–22. doi: 10.1016/S0378-5122(96)01063-8
121. Scanlan JM, Werner JJ, Legg RL, Laudenslager ML. Natural killer cell activity is reduced in association with oral contraceptive use. *Psychoneuroendocrinology.* (1995) 20:281–7. doi: 10.1016/0306-4530(94)00059-J
122. Hanna N, Schneider M. Enhancement of tumor metastasis and suppression of natural killer cell activity by beta-estradiol treatment. *J Immunol.* (1983) 130:974–80.
123. Kalland T, Forsberg JG. Natural killer cell activity and tumor susceptibility in female mice treated neonatally with diethylstilbestrol. *Cancer Res.* (1981) 41(12 Pt 1):5134–40.
124. Screpanti I, Santoni A, Gulino A, Herberman RB, Frati L. Estrogen and antiestrogen modulation of the levels of mouse natural killer activity and large granular lymphocytes. *Cell Immunol.* (1987) 106:191–202. doi: 10.1016/0008-8749(87)90163-8
125. Jiang X, Orr BA, Kranz DM, Shapiro DJ. Estrogen induction of the granzyme B inhibitor, proteinase inhibitor 9, protects cells against apoptosis mediated by cytotoxic T lymphocytes and natural killer cells. *Endocrinology.* (2006) 147:1419–26. doi: 10.1210/en.2005-0996
126. Liu C, Yu S, Zinn K, Wang J, Zhang L, Jia Y, et al. Murine mammary carcinoma exosomes promote tumor growth by suppression of NK cell function. *J Immunol.* (2006) 176:1375–85. doi: 10.4049/jimmunol.176.3.1375
127. Hoffman W, Lakkis FG, Chalasani G. B cells, antibodies, and more. *Clin J Am Soc Nephrol.* (2016) 11:137–54. doi: 10.2215/CJN.09430915
128. Grimaldi CM, Cleary J, Dagtas AS, Moussai D, Diamond B. Estrogen alters thresholds for B cell apoptosis and activation. *J Clin Invest.* (2002) 109:1625–33. doi: 10.1172/JCI0214873
129. Kanda N, Tamaki K. Estrogen enhances immunoglobulin production by human PBMCs. *J Allergy Clin Immunol.* (1999) 103(2 Pt 1):282–8. doi: 10.1016/S0091-6749(99)70503-8
130. Paavonen T, Andersson LC, Adlercreutz H. Sex hormone regulation of in vitro immune response. Estradiol enhances human B cell maturation via inhibition of suppressor T cells in pokeweed mitogen-stimulated cultures. *J Exp Med.* (1981) 154:1935–45. doi: 10.1084/jem.154.6.1935
131. Myers MJ, Petersen BH. Estradiol induced alterations of the immune system–I. Enhancement of IgM production. *Int J Immunopharmacol.* (1985) 7:207–13. doi: 10.1016/0192-0561(85)90028-1
132. Vollmers HP, Brandlein S. Natural antibodies and cancer. *N Biotechnol.* (2009) 25:294–8. doi: 10.1016/j.nbt.2009.03.016
133. Diaz-Zaragoza M, Hernandez-Avila R, Viedma-Rodriguez R, Arenas-Aranda D, Ostoa-Saloma P. Natural and adaptive IgM antibodies in the recognition of tumor-associated antigens of breast cancer (Review). *Oncol Rep.* (2015) 34:1106–14. doi: 10.3892/or.2015.4095
134. Papatestas AE, Bramis J, Aufses AH Jr. Serum immunoglobulins in women with breast cancer. *J Surg Oncol.* (1979) 12:155–63. doi: 10.1002/jso.2930120209
135. Catusse J, Wollner S, Leick M, Schrottner P, Schraufstatter I, Burger M. Attenuation of CXCR4 responses by CCL18 in acute lymphocytic leukemia B cells. *J Cell Physiol.* (2010) 225:792–800. doi: 10.1002/jcp.22284
136. Nugent A, Proia RL. The role of G protein-coupled receptors in lymphoid malignancies. *Cell Signal.* (2017) 39:95–107. doi: 10.1016/j.cellsig.2017.08.002
137. Zhu J, Paul WE. CD4 T cells: fates, functions, and faults. *Blood.* (2008) 112:1557–69. doi: 10.1182/blood-2008-05-078154
138. Okasha SA, Ryu S, Do Y, McKallip RJ, Nagarkatti M, Nagarkatti PS. Evidence for estradiol-induced apoptosis and dysregulated T cell maturation in the thymus. *Toxicology.* (2001) 163:49–62. doi: 10.1016/S0300-483X(01)00374-2
139. Staples JE, Gasiewicz TA, Fiore NC, Lubahn DB, Korach KS, Silverstone AE. Estrogen receptor alpha is necessary in thymic development and estradiol-induced thymic alterations. *J Immunol.* (1999) 163:4168–74.
140. Erlandsson MC, Ohlsson C, Gustafsson JA, Carlsten H. Role of oestrogen receptors alpha and beta in immune organ development and in oestrogen-mediated effects on thymus. *Immunology.* (2001) 103:17–25. doi: 10.1046/j.1365-2567.2001.01212.x
141. Lelu K, Laffont S, Delpy L, Paulet PE, Perinat T, Tschanz SA, et al. Estrogen receptor alpha signaling in T lymphocytes is required for estradiol-mediated inhibition of Th1 and Th17 cell differentiation and protection against experimental autoimmune encephalomyelitis. *J Immunol.* (2011) 187:2386–93. doi: 10.4049/jimmunol.1101578
142. Michalek RD, Gerriets VA, Jacobs SR, Macintyre AN, MacIver NJ, Mason EF, et al. Cutting edge: distinct glycolytic and lipid oxidative metabolic programs are essential for effector and regulatory CD4+ T cell subsets. *J Immunol.* (2011) 186:3299–303. doi: 10.4049/jimmunol.1003613
143. Michalek RD, Gerriets VA, Nichols AG, Inoue M, Kazmin D, Chang CY, et al. Estrogen-related receptor-alpha is a metabolic regulator of effector T-cell activation and differentiation. *Proc Natl Acad Sci USA.* (2011) 108:18348–53. doi: 10.1073/pnas.1108856108
144. Grasso G, Muscettola M. The influence of beta-estradiol and progesterone on interferon gamma production *in vitro*. *Int J Neurosci.* (1990) 51:315–7. doi: 10.3109/00207459008999730
145. Karpuzoglu-Sahin E, Hissong BD, Ansar Ahmed S. Interferon-gamma levels are upregulated by 17-beta-estradiol and diethylstilbestrol. *J Reprod Immunol.* (2001) 52:113–27. doi: 10.1016/S0165-0378(01)00117-6
146. Salem ML. Estrogen, a double-edged sword: modulation of TH1- and TH2-mediated inflammations by differential regulation of TH1/TH2 cytokine production. *Curr Drug Targets Inflamm Allergy.* (2004) 3:97–104. doi: 10.2174/1568010043483944
147. Malutan AM, Dan M, Nicolae C, Carmen M. Proinflammatory and anti-inflammatory cytokine changes related to menopause. *Prz Menopauzalny.* (2014) 13:162–8. doi: 10.5114/pm.2014.43818
148. Kamada M, Irahara M, Maegawa M, Ohmoto Y, Murata K, Yasui T, et al. Transient increase in the levels of T-helper 1 cytokines in postmenopausal women and the effects of hormone replacement therapy. *Gynecol Obstet Invest.* (2001) 52:82–8. doi: 10.1159/000052948
149. Salem ML, Matsuzaki G, Madkour GA, Nomoto K. Beta-estradiol-induced decrease in IL-12 and TNF-alpha expression suppresses macrophage functions in the course of Listeria monocytogenes infection in mice. *Int J Immunopharmacol.* (1999) 21:481–97. doi: 10.1016/S0192-0561(99)00027-2
150. Lambert KC, Curran EM, Judy BM, Milligan GN, Lubahn DB, Estes DM. Estrogen receptor alpha (ERalpha) deficiency in macrophages results in increased stimulation of CD4+ T cells while 17beta-estradiol acts through ERalpha to increase IL-4 and GATA-3 expression in CD4+ T cells independent of antigen presentation. *J Immunol.* (2005) 175:5716–23. doi: 10.4049/jimmunol.175.9.5716
151. Sakaguchi S, Ono M, Setoguchi R, Yagi H, Hori S, Fehervari Z, et al. Foxp3+ CD25+ CD4+ natural regulatory T cells in dominant self-tolerance and autoimmune disease. *Immunol Rev.* (2006) 212:8–27. doi: 10.1111/j.0105-2896.2006.00427.x
152. Chaudhary B, Elkord E. Regulatory T cells in the tumor microenvironment and cancer progression: role and therapeutic targeting. *Vaccines.* (2016) 4:E28. doi: 10.3390/vaccines4030028
153. Polanczyk MJ, Carson BD, Subramanian S, Afentoulis M, Vandenbark AA, Ziegler SF, et al. Cutting edge: estrogen drives expansion of the CD4+CD25+ regulatory T cell compartment. *J Immunol.* (2004) 173:2227–30. doi: 10.4049/jimmunol.173.4.2227
154. Tai P, Wang J, Jin H, Song X, Yan J, Kang Y, et al. Induction of regulatory T cells by physiological level estrogen. *J Cell Physiol.* (2008) 214:456–64. doi: 10.1002/jcp.21221
155. Polanczyk MJ, Hopke C, Vandenbark AA, Offner H. Treg suppressive activity involves estrogen-dependent expression of programmed death-1 (PD-1). *Int Immunol.* (2007) 19:337–43. doi: 10.1093/intimm/dxl151
156. Prieto GA, Rosenstein Y. Oestradiol potentiates the suppressive function of human CD4 CD25 regulatory T cells by promoting their proliferation. *Immunology.* (2006) 118:58–65. doi: 10.1111/j.1365-2567.2006.02339.x
157. Adurthi S, Kumar MM, Vinodkumar HS, Mukherjee G, Krishnamurthy H, Acharya KK, et al. Oestrogen receptor-alpha binds the FOXP3 promoter and modulates regulatory T-cell function in human cervical cancer. *Sci Rep.* (2017) 7:17289. doi: 10.1038/s41598-017-17102-w
158. Brunsing RL, Owens KS, Prossnitz ER. The G protein-coupled estrogen receptor (GPER) agonist G-1 expands the regulatory T-cell population under TH17-polarizing conditions. *J Immunother.* (2013) 36:190–6. doi: 10.1097/CJI.0b013e31828d8e3b

159. Askenasy N, Kaminitz A, Yarkoni S. Mechanisms of T regulatory cell function. *Autoimmun Rev.* (2008) 7:370–5. doi: 10.1016/j.autrev.2008.03.001
160. Luo CY, Wang L, Sun C, Li DJ. Estrogen enhances the functions of CD4(+)CD25(+)Foxp3(+) regulatory T cells that suppress osteoclast differentiation and bone resorption in vitro. *Cell Mol Immunol.* (2011) 8:50–8. doi: 10.1038/cmi.2010.54
161. Tong CWS, Wu M, Cho WCS, To KKW. Recent advances in the treatment of breast cancer. *Front Oncol.* (2018) 8:227. doi: 10.3389/fonc.2018.00227
162. Li Z, Dong P, Ren M, Song Y, Qian X, Yang Y, et al. PD-L1 expression is associated with tumor FOXP3(+) regulatory T-cell infiltration of breast cancer and poor prognosis of patient. *J Cancer.* (2016) 7:784–93. doi: 10.7150/jca.14549
163. Generali D, Bates G, Berruti A, Brizzi MP, Campo L, Bonardi S, et al. Immunomodulation of FOXP3+ regulatory T cells by the aromatase inhibitor letrozole in breast cancer patients. *Clin Cancer Res.* (2009) 15:1046–51. doi: 10.1158/1078-0432.CCR-08-1507
164. Berry J, Green BJ, Matheson DS. Modulation of natural killer cell activity by tamoxifen in stage I post-menopausal breast cancer. *Eur J Cancer Clin Oncol.* (1987) 23:517–20. doi: 10.1016/0277-5379(87)90313-0
165. Jiang X, Shapiro DJ. The immune system and inflammation in breast cancer. *Mol Cell Endocrinol.* (2014) 382:673–82. doi: 10.1016/j.mce.2013.06.003
166. Karpuzoglu E, Fenaux JB, Phillips RA, Lengi AJ, Elvinger FO, Ansar Ahmed S. Estrogen up-regulates inducible nitric oxide synthase, nitric oxide, and Cyclooxygenase-2 in splenocytes activated with T cell stimulants: role of interferon-γ. *Endocrinology.* (2006) 147:662–71. doi: 10.1210/en.2005-0829
167. Rath M, Muller I, Kropf P, Closs EI, Munder M. Metabolism via arginase or nitric oxide synthase: two competing arginine pathways in macrophages. *Front Immunol.* (2014) 5:532. doi: 10.3389/fimmu.2014.00532
168. Suzuki M, Fujimura T, Enomoto Y, Nishimatsu H, Ishikawa A. 17β-estradiol-mediated elevation of peripheral white blood cell count during estramustine phosphate therapy for prostate cancer. *Int J Endocrinol Metab.* (2011) 9:347–51. doi: 10.5812/Kowsar.1726913X.2246
169. Harrison JD, Watson S, Ellis IO, Morris DL. A study on the effects of estradiol and estramustine on gastrointestinal cell lines. *Eur J Surg Oncol.* (1990) 16:116–20.
170. Flores R, Dohrmann S, Schaal C, Hakkim A, Nizet V, Corriden R. The selective estrogen receptor modulator raloxifene inhibits neutrophil extracellular trap formation. *Front Immunol.* (2016) 7:566. doi: 10.3389/fimmu.2016.00566
171. Corriden R, Hollands A, Olson J, Derieux J, Lopez J, Chang JT, et al. Tamoxifen augments the innate immune function of neutrophils through modulation of intracellular ceramide. *Nat Commun.* (2015) 6:8369. doi: 10.1038/ncomms9369
172. Palacios-Arreola MI, Nava-Castro KE, Rio-Araiza VHD, Perez-Sanchez NY, Morales-Montor J. A single neonatal administration of Bisphenol A induces higher tumour weight associated to changes in tumour microenvironment in the adulthood. *Sci Rep.* (2017) 7:10573. doi: 10.1038/s41598-017-10135-1
173. Denkert C, Wienert S, Poterie A, Loibl S, Budczies J, Badve S, et al. Standardized evaluation of tumor-infiltrating lymphocytes in breast cancer: results of the ring studies of the international immuno-oncology biomarker working group. *Mod Pathol.* (2016) 29:1155–64. doi: 10.1038/modpathol.2016.109
174. Yuan Y. Modelling the spatial heterogeneity and molecular correlates of lymphocytic infiltration in triple-negative breast cancer. *J R Soc Interface.* (2015) 12:1–13. doi: 10.1098/rsif.2014.1153
175. Savas P, Salgado R, Denkert C, Sotiriou C, Darcy PK, Smyth MJ, et al. Clinical relevance of host immunity in breast cancer: from TILs to the clinic. *Nat Rev Clin Oncol.* (2016) 13:228–41. doi: 10.1038/nrclinonc.2015.215
176. Bianchini G, Gianni L. The immune system and response to HER2-targeted treatment in breast cancer. *Lancet Oncol.* (2014) 15:e58–68. doi: 10.1016/S1470-2045(13)70477-7
177. West NR, Milne K, Truong PT, Macpherson N, Nelson BH, Watson PH. Tumor-infiltrating lymphocytes predict response to anthracycline-based chemotherapy in estrogen receptor-negative breast cancer. *Breast Cancer Res.* (2011) 13:R126. doi: 10.1186/bcr3072
178. Takagi K, Ishida T, Miki Y, Hirakawa H, Kakugawa Y, Amano G, et al. Intratumoral concentration of estrogens and clinicopathological changes in ductal carcinoma *in situ* following aromatase inhibitor letrozole treatment. *Br J Cancer.* (2013) 109:100–8. doi: 10.1038/bjc.2013.284
179. Suzuki T, Miki Y, Akahira J, Moriya T, Ohuchi N, Sasano H. Aromatase in human breast carcinoma as a key regulator of intratumoral sex steroid concentrations. *Endocr J.* (2008) 55:455–63. doi: 10.1507/endocrj.K07E-053
180. Marabelle A, Andtbacka R, Harrington K, Melero I, Leidner R, de Baere T, et al. Starting the fight in the tumor: expert recommendations for the development of human intratumoral immunotherapy (HIT-IT). *Ann Oncol.* (2018) 29:2163–74. doi: 10.1093/annonc/mdy423

**Conflict of Interest Statement:** The authors declare that the research was conducted in the absence of any commercial or financial relationships that could be construed as a potential conflict of interest.

*Copyright © 2019 Segovia-Mendoza and Morales-Montor. This is an open-access article distributed under the terms of the Creative Commons Attribution License (CC BY). The use, distribution or reproduction in other forums is permitted, provided the original author(s) and the copyright owner(s) are credited and that the original publication in this journal is cited, in accordance with accepted academic practice. No use, distribution or reproduction is permitted which does not comply with these terms.*
